# The activation of mTOR signalling modulates DNA methylation by enhancing DNMT1 translation in hepatocellular carcinoma

**DOI:** 10.1186/s12967-023-04103-9

**Published:** 2023-04-23

**Authors:** Mengke Chen, Yi Fang, Meinong Liang, Ning Zhang, Xinyue Zhang, Lixia Xu, Xuxin Ren, Qingfeng Zhang, Yufeng Zhou, Sui Peng, Jun Yu, Judeng Zeng, Xiaoxing Li

**Affiliations:** 1 Department of Oncology, Sun Yat-Sen University First Affiliated Hospital, Guangzhou, China; 2 Institute of Precision Medicine, Sun Yat-Sen University First Affiliated Hospital, Guangzhou, China; 3 Department of Gastroenterology, Sun Yat-Sen University First Affiliated Hospital, Guangzhou, China; 4 Sun Yat-Sen University Cancer Center, Guangzhou, China; 5 Department of Anaesthesia and Intensive Care, The Chinese University of Hong Kong, Hong Kong, China

**Keywords:** Hepatocellular carcinoma, Protein translation, DNA methylation, DNMT1, Pyrimidine rich translational element

## Abstract

**Background:**

Both dysregulation of mechanistic target of rapamycin (mTOR) signalling and DNA methylation patterns have been shown to be closely associated with tumor progression and serve as promising targets for hepatocellular carcinoma (HCC) therapy. Although their respective roles in HCC have been extensively revealed, the existence of molecular interactions between them remains largely unknown.

**Methods:**

The association of DNA methylation and mTOR signalling in HCC tissues and cell lines was assessed. A Kaplan‒Meier analysis was applied to estimate the overall survival (OS) and recurrence-free survival (RFS) of HCC patients. The modulation of DNMT1 by mTOR in HCC cell lines was determined. The effect of the drug combination in cell lines and mouse models was examined.

**Results:**

The results showed that the DNA methylation level was positively associated with the activation of mTOR signalling in HCC tissues and cell lines. Moreover, HCC patients with higher DNA methylation levels and enhanced activation of mTOR signalling exhibited the worst prognosis. Then, we screened methylation-related enzymes and found that the activation of mTOR signalling increased DNMT1 expression and activity. In addition, mTOR enhanced the translational efficiency of DNMT1 in a 4E-BP1-dependent manner, which is based on the pyrimidine rich translational element (PRTE)-containing 5′UTR of DNMT1. Moreover, we demonstrated that the combined inhibition of mTOR and DNMT synergistically inhibited HCC growth in vitro and in vivo.

**Conclusions:**

In addition to some already identified pro-cancer downstream molecules, the activation of mTOR signalling was found to promote DNA methylation by increasing the translation of DNMT1. Furthermore, combined targeting of mTOR and DNMT1 has been demonstrated to have a more effective tumor suppressive function in HCC.

**Supplementary Information:**

The online version contains supplementary material available at 10.1186/s12967-023-04103-9.

## Introduction

Hepatocellular carcinoma (HCC) is one of the most prevalent malignancies worldwide, but effective treatments for patients in advanced stages are lacking [1]. The multikinase inhibitor sorafenib induces limited improvement in overall survival [2]. Regorafenib has been approved for advanced HCC but is used only as a second-line therapy after sorafenib failure [3]. However, the high frequency of drug resistance and serious side effects still restrict the clinical applications of these drugs [4]. Thus, the exploitation of more effective therapy for advanced HCC remains urgent.

As the major metabolic organ in humans, the liver performs a series of metabolic tasks. Abnormal liver metabolism can lead to liver disease and even HCC. Aberrant activation of the signalling pathway downstream of mechanistic target of rapamycin (mTOR), the critical sensor of environmental metabolic signals, is also involved in the malignant progression of HCC [5]. mTOR acts as a core serine/threonine kinase in two multicomponent complexes, mTORC1 and mTORC2 [6]. Although these complexes share several subunits and functional characteristics, they show distinct sensitivities to rapamycin, a selective mTOR inhibitor. In addition, mTORC1 is a crucial sensor of various environmental cues, including growth factors, energy status, amino acid levels, and stress, to control cell growth and metabolism mainly by regulating the phosphorylation of two well-studied substrates, eukaryotic translation initiation factor 4E (eIF4E) binding protein 1 (4E-BP1) and ribosomal protein S6 kinase (S6K) (6). The mTORC1 pathway is activated in a wide spectrum of cancers, mainly due to frequent activating mutations in upstream factors, such as PI3KCA, RAS, and RAF, as well as loss-of-function mutations in related tumour suppressors, such as PTEN and TSC1/2 [7, 8]. The frequent hyperactivation of the mTOR pathway makes it a promising target for cancer therapy. Indeed, first-generation rapalogs, including temsirolimus and everolimus, have been approved by the FDA for renal and breast cancer therapy [9]. Additionally, many clinical trials based on second-generation mTOR inhibitors for cancer therapy are ongoing [10]. However, the limited clinical response rates to mTOR-targeted therapy and the development of drug resistance remain major challenges [11, 12]. A previous genomic study of relapsed cases clarified that activating mutations and gene copy number gain or loss are the main reasons for therapeutic failure [13]. Therefore, researchers have continued to develop novel mTOR inhibitors and regimens to overcome the established resistance [14].

In addition to genetic mechanisms, epigenetic alterations, which can mediate cell status transitions during tumorigenesis [15] and facilitate resistance to the cytotoxic effects of drugs during antitumour therapy [16], have also attracted great attention. DNA methylation plays a critical role in carcinogenesis by regulating the expression of essential factors that promote the formation of neoplastic features. In turn, DNA methylation is dynamic during oncotherapy, such as targeted therapy with mTOR inhibitors, as exemplified by a recent report indicating that the DNA methylation profile was changed in cells with LKB1 loss and KRAS activating mutations via mTORC1-dependent activation of serine–glycine–one-carbon metabolism [17]. This evidence suggests that mTORC1 can function as a DNA methylation regulator. However, the exact mechanism by which mTORC1 regulates DNA methylation remains largely unexplored. Determining the mechanism underlying the interplay between the mTOR signalling pathway and DNA methylation may help us to better estimate the therapeutic effect of mTOR inhibitors in cancers, including HCC.

In the present study, we discovered that mTORC1 could modulate DNA methylation patterns by regulating the translational efficiency of DNA methyltransferase 1 (DNMT1) in a 4E-BP1-dependent manner in HCC. Furthermore, we evaluated the therapeutic effects of combination treatment with mTOR and DNA methylation inhibitors in vitro and in vivo.

## Materials and methods

### Patients and specimens

Fifty-two paraffin-embedded samples with complete follow-up data were collected from HCC patients who underwent surgery at The First Affiliated Hospital of Sun Yat-sen University. Overall survival (OS) was calculated from the date of surgery to either death or the final follow-up. Recurrence-free survival (RFS) was defined as the interval from the time of surgery to either recurrence or the last follow-up. This study was reviewed and approved by the ethics committees of the First Affiliated Hospital of Sun Yat-sen University. The pathological information of the patients is listed in Additional file 1: Table S1.

### Cell lines and cell culture

The SNU423, SNU449, and MHCC-97H cell lines were purchased from ATCC. The WT and TSC1^–/–^ MEF lines were gifts from X.F. Steven Zheng. SNU423 and SNU449 cells were cultured in RPMI 1640 medium (Gibco, USA), while MHCC-97H cells and MEFs were maintained in high-glucose Dulbecco’s modified Eagle’s medium (DMEM; Gibco). Fetal bovine serum (FBS; 10%, Gibco) and penicillin–streptomycin solution were added to the medium during cell culture. The cells were incubated at 37 °C in a humidified chamber containing 5% CO_2_. Rapamycin, decitabine, and MG-132 were purchased from Selleck Chemicals (USA); CHX was purchased from Sigma (USA).

### Plasmids and transfection

The 4E-BP1 expression plasmid was generated by inserting the synthetic DNA fragment (NM_004095) into the empty backbone of pcDNA3.1. In 4E-BP1-4A, a mutant plasmid, Thr37, Thr46, Ser65, and Thr70 were replaced by Ala. The DNMT1 5′UTR (chr19:10305576-10305755) fragment was cloned by PCR, and the pGL3 control vector was digested with restriction enzymes (HindIII and NcoI). Deletion of specific fragments of the DNMT1-5′UTR was performed by designing different primer pairs, and fragments were linked by fusion (Takara, In-Fusion HD Cloning Kits) (see detailed methods in the Additional file 1). The plasmids with the 14-base mutation in the 5′UTR and deletion of 5′UTR fragments were constructed by inserting the corresponding synthetic DNA fragments into the vector. Plasmids were transfected into cells using Lipofectamine 3000 (Invitrogen, USA) according to the manufacturer’s instructions.

### Genomic DNA extraction and dot blot analysis

DNA was extracted from cells using a TIANamp Genomic DNA Kit (Tiangen) according to the manufacturer’s instructions. DNA was sonicated to generate fragments between 200 and 500 bp. Fragmented genomic DNA was diluted to 50 ng/μl in 100 μl of nuclease-free water. Then, DNA was denatured by adding 100 μl of 2 × DNA denaturing buffer (200 mM NaOH and 20 mM EDTA) and incubated at 95 °C for 10 min. Then, 200 μl of 20 × SSC buffer [3.0 M NaCl, 0.3 M sodium citrate (pH 7.0)] was added, and the tube was immediately placed on ice for 5 min. Then, the appropriate volume of nuclease-free water was added to bring the DNA solution to a proper concentration. A twofold dilution of the final DNA solution was added to separate wells of the 96-well dot blot apparatus. The nylon membrane was removed from the 96-well dot blot apparatus, dried at 60 °C for one hour, and crosslinked by UV irradiation at 1200 J/m^2^. The subsequent procedures were performed in accordance with the protocol for Western blotting.

### DNMT1 activity assay

Nuclear protein was extracted with a Nuclear Extraction Kit (Abcam, ab113474), and the protein concentration was determined with Bradford Reagent (Abcam, ab119216). DNMT1 enzymatic activity in cells was evaluated using a DNMT1 assay kit (Abcam, ab113469). The assay was performed according to the manufacturer’s instructions, and 10 µg of nuclear protein was used in each test.

### Polysome fractionation and analysis

One 15 cm dish of cells (approximately 1 × 10^7^ cells) was prepared and treated with 100 μg/ml CHX for 10 min prior to lysis. After two washes with ice-cold PBS (with 100 μg/ml cycloheximide), cells were collected by centrifugation (4 °C, 500 × *g*, 5 min). Cells were resuspended in lysis buffer [5 mM Tris–HCl (pH 7.5), 2.5 mM MgCl_2_, 1.5 mM KCl and 1 × EDTA-free protease inhibitor cocktail], and CHX, dithiothreitol (DTT), and an RNase inhibitor (Promega) were added and vortexed for 5 s. The lysates were then centrifuged, and the supernatant (~ 500 μl) was transferred to a new prechilled 1.5 ml tube. Ten percent (v/v) of the lysate was kept as input for determining cytosolic steady-state mRNA levels. Lysates were layered onto 5–50% sucrose density gradients made by the manufacturer. The gradients were centrifuged in an SW-41Ti rotor at 32,000 rpm and 4 °C for 2 h and were then sampled using a Labconco Auto Densi-Flow Gradient Fractionator connected to an ISCO Tris pump with constant monitoring of the OD254 value.

Approximately 10 fractions were collected in total, and 0.5 ml of TRIzol and 5 ng of polyA + synthetic luciferase mRNA (Promega) were added to each fraction. RNA was extracted from the diluted fractions, and cDNA was prepared using Superscript III Reverse Transcriptase (Invitrogen) according to the manufacturer’s instructions. The transcript abundance was determined by qPCR using SYBR Green PCR Mix (Applied Biosystems). Transcript abundances were then normalized to luciferase abundances.

### Luciferase reporter assay

Firefly luciferase and Renilla luciferase pGL3 vectors were transfected into cells at a 20:1 ratio in a 24-well plate. Rapamycin was added to the medium after 24 h, and cells were harvested at 48 h. Firefly and Renilla luciferase signals were detected with a Promega GloMax 20/20 luminometer according to the manufacturer’s instructions (Promega, Dual-Luciferase^®^ Reporter Assay System). In addition, RNA was extracted from cells, and firefly and Renilla mRNA expression levels were measured. The translational efficiency of the 5′UTR was calculated by normalizing the firefly luciferase level to the Renilla luciferase level.

### RNA interference

Small interfering RNA (siRNA) was purchased from Shanghai GenePharma Co., Ltd. and transfected into cells using Lipofectamine 3000 (Invitrogen, USA) according to the manufacturer’s instructions. The siRNA sequences are listed in Additional file 1: Table S2.

### RNA extraction and RT–qPCR

RNA was extracted from cells using TRIzol (Invitrogen, USA). cDNA was synthesized using the SuperScript™ IV One-Step RT–PCR System (TaKaRa). Quantitative PCR (qPCR) was performed in triplicate, and relative expression levels were calculated by the 2^−ΔΔCt^ method with normalization to *ACTB* levels. The primer sequences are listed in Additional file 1: Table S2.

### Immunofluorescence

Cells were cultured in µ-Slide 8 Well chambers (IBIDI, Germany) and were then fixed with 4% formaldehyde at room temperature (RT) for 40 min. After blocking with PBS containing 4% bovine serum albumin (BSA) (Sigma, USA) for 90 min, cells were incubated first with an anti-5mC antibody (Abcam, ab10805) and then with an Alexa Fluor 488-conjugated secondary antibody (Life Technologies, USA). Nuclei were stained using 4′,6-diamidino-2-phenylindole dihydrochloride (DAPI) (Life Technologies, USA). Images were acquired in multitracking mode using an Olympus scanning confocal microscope (FV1000, Olympus, Japan). An analysis of IF was performed as previously described [18].

### Western blot analysis

Cells were lysed with RIPA buffer (CST) containing protease and phosphatase inhibitor cocktails (KeyGEN) on ice. The supernatant was collected after centrifugation, and the concentration was determined by a BCA assay (Thermo Fisher Scientific, USA). A total of 30 μg of protein extract was separated by sodium dodecyl sulfate–polyacrylamide gel electrophoresis (SDS‒PAGE) and transferred onto a polyvinylidene fluoride (PVDF) membrane. The membrane was blocked with 5% non-fat milk in TBST buffer [10 mM Tris–HCl (pH 7.4) containing 0.1% Tween 20] and was then incubated with a primary antibody at 4 °C overnight (Additional file 1: Table S3). After washing 3 times, the membrane was incubated with a secondary antibody (CST) at room temperature for 40 min, and immunoreactions were detected with a chemiluminescent substrate (Thermo). Images were acquired with a VersaDoc 3000 Imaging System (Bio-Rad), and densitometric analysis was performed with ImageJ (National Institutes of Health, Bethesda, MD). The relative protein expression was calculated as a ratio of the density of each protein band relative to the loading control GAPDH and was normalized to unstimulated controls.

### Cell proliferation and colony formation assays

The cell proliferation assay was performed according to the CCK-8 assay (APExBIO, USA) manufacturer’s instructions. HCC cells were seeded into 96-well plates (1000 cells per well) and incubated overnight. The indicated concentrations of drugs were added, and the optical density (OD) was measured at 450 nm (OD450). The OD450 was then measured daily. For the colony formation assay, 800 HCC cells were seeded into 6-well plates and cultured overnight. Then, drugs were added at the indicated concentrations. Two weeks later, colonies were fixed with methyl alcohol and counted after staining with 1% Crystal Violet Staining Solution (Beyotime).

### Immunohistochemistry (IHC)

IHC was conducted using protocols described previously [19]. Briefly, sections were incubated with primary antibodies against 5mC (ab10805, Abcam, Cambridge, UK), p-mTOR (ab109268, Abcam), DNMT1(5032S, CST, Danvers) and Ki67 (ab1667, Abcam). The immunostaining intensity was given a score of 1–3 (1, negative or weak; 2, moderate; 3, strong), and the percentage of positive immunostaining was scored as 0–100%. The percentage and intensity score were multiplied to obtain the total immunohistochemistry score (range of 0–300) of 5mC and p-mTOR. The IHC score of DNMT1 and Ki67 were obtained by multiplying the percentage of positive cells and 100 (range of 0–100).

### Animal study

The animal study was approved by the Institutional Animal Care and Use Committee of Sun Yat-Sen University. MHCC-97H cells (1 × 10^7^) were injected into the right flanks of female nude mice (aged 4 weeks). When the tumour volume was 500 mm^3^, the tumours were cut into 1-mm^3^ pieces. In the subcutaneous tumour model, tumour fragments were implanted into the right flanks of female nude mice (4 weeks old, n = 20). In the in situ tumour model, a tumour fragment was inserted into a small incision in the liver of each mouse. After 10 days, when the average subcutaneous tumour size was 100 mm^3^, the mice (n = 6 per group) were randomized into four groups. Decitabine (1 mg/kg) was solubilized in a 100-μl volume of PBS. Rapamycin (1 mg/kg) was dissolved in 100 μl of 30% polyethylene glycol 300 (PEG 300) and 5% Tween 80. These drugs were administered intraperitoneally daily and freshly prepared just before administration. The mice in the control group were administered the vehicle used for solubilization. They were sacrificed at humane endpoint. The tumours were measured daily, and the tumour volumes were calculated with the following equation: Tumour volume = Length $\times$ Width^2^/($\pi$/6). Overall survival (OS) was defined as the interval between the time of administration and death.

### Statistical analysis

All statistical tests were performed using SPSS (version 11.0; SPSS, Inc., Chicago, IL, USA) or GraphPad Software (version 5.0 for Windows; GraphPad Prism Inc., San Diego, CA, USA). Survival curves were obtained using the Kaplan‒Meier method. Subgroups of each immunostaining parameter were divided by the median values. An analysis of the association between continuous variables was conducted using Spearman’s rank correlation coefficient. Differences between two groups were assessed by the two-tailed Student’s t test, and comparisons among multiple groups were analysed using one-way ANOVA, followed by Tukey’s post hoc tests. The data were presented as the mean ± SD values. *P* values < 0.05 were considered to indicate statistical significance.

## Results

### The cellular DNA methylation profile was modulated by the mTOR signalling pathway

**Fig. 1:**
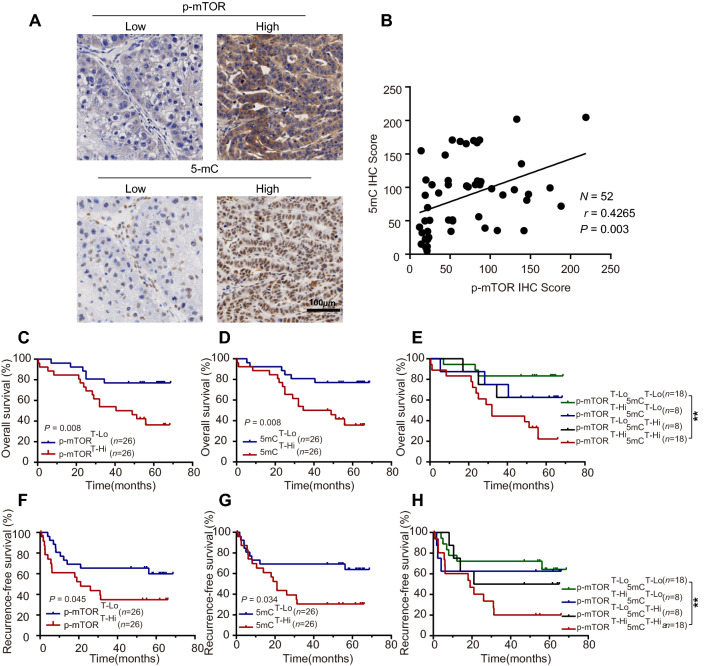
Aberrant activation of mTOR signalling synergizes with DNA methylation to promote tumour progression in HCC patients. **A** Representative IHC images demonstrate low (left) and high (right) scores for p-mTOR (above) and 5mC (under) in tumoral regions, the low or high images of p-mTOR and 5mC were derived from the same patient. Scale bar = 100 μm. **B** Linear correlation between p-mTOR IHC scores and 5mC IHC scores. Correlations were analysed by Spearman’s rank correlation coefficient test. **C–E** Cumulative overall survival curves of HCC patients based on p-mTOR and 5mC. **F–H** Recurrence-free survival curves of HCC patients based on p-mTOR and 5mC. Overall survival and recurrence-free survival were estimated using the Kaplan–Meier method and compared using the log‒rank test. The patients were divided into two groups according to the median IHC score of p-mTOR and 5mC. **P < 0.01. T, intra-tumoral region; lo, low; hi, high

To investigate the relationship between the mTOR signalling pathway and DNA methylation, immunohistochemistry (IHC) staining was performed with 52 paired HCC and adjacent tissues. As shown in Fig. 1A and Additional file 1: Fig. S1A, patients were divided into two groups based on the median value of the IHC score for the phosphorylation of mTOR (p-mTOR) or 5-methylcytosine (5mC) in tumoral or adjacent regions. The p-mTOR level was significantly increased in HCC tissues compared to adjacent tissues (*P* = 0.004) and no significant difference was observed in the 5mC level between HCC and adjacent tissues (Additional file 1: Fig. S1B). However, the 5mC level was positively correlated with the p-mTOR level in tumoral regions (*P* = 0.003, Fig. 1B). Similar correlations were not observed in adjacent tissues (Additional file 1: Fig. S1C). Moreover, the relationship between the 5mC/p-mTOR levels and patient survival was further investigated. Negative correlations were detected between the intratumor p-mTOR level and overall survival (OS, *P* = 0.008; Fig. 1C) and recurrence-free survival (RFS, *P* = 0.045; Fig. 1F). Similarly, a high level of intratumor 5mC predicted poor survival (OS: *P* = 0.008, Fig. 1D; RFS: *P* = 0.034, Fig. 1G). In addition, patients in the p-mTOR^high^ and 5mC^high^ groups exhibited the worst OS (5-year OS rate: 22.2%) and RFS (5-year RFS rate: 20.1%) compared with those in the p-mTOR^high^ and 5mC^low^ group (5-year OS rate: 62.5%; 5-year RFS rate: 62.5%), the p-mTOR^low^ and 5mC^high^ group (5-year OS rate: 62.5%; 5-year RFS rate: 50.0%) and the p-mTOR^low^ and 5mC^low^ group (5-year OS rate: 83.3%; 5-year RFS rate: 64.2%; Fig. 1E, H). Similar trends were not observed in adjacent normal tissues (Additional file 1: Fig. S1D–I).

**Fig. 2:**
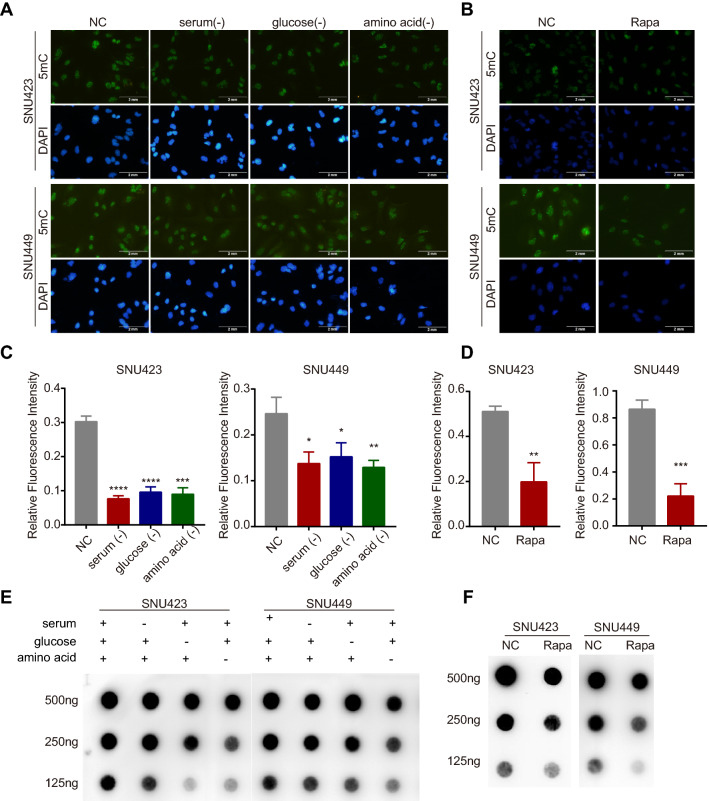
Repression of the mTOR signalling pathway reduces DNA methylation in liver cancer cells. **A**, **C** IF staining and quantitative analysis of 5mC in SNU423 and SNU449 cells; nuclei were stained with DAPI (scale bar, 2 mm). Cells in the control group were cultured in complete medium, whereas cells in the experimental group were subjected to starvation (cultured in complete medium without serum, glucose, and amino acids) for 24 h. **B**, **D** IF staining and quantitative analysis for 5mC in SNU423 and SNU449 cells treated with rapamycin (500 nM) for 24 h. **E**, **F** Dot blot analysis of the 5mC levels in SNU423 and SNU449 cells treated with starvation or rapamycin (500 nM) for 24 h. 500 ng, 250 ng and 125 ng meaned the loading amount of cellular DNA

Besides, we performed 5-methylcytosine (5mC)-specific immunofluorescence (IF) in HCC cell lines and found that the 5mC level was significantly decreased in HCC cells exposed to a nutrient-deficient environment, in which mTOR signalling was suppressed (Fig. 2A, C). Consistent with the results of nutrient starvation, inhibition of the mTOR signalling pathway by rapamycin also induced a significant decline in the 5mC level (Fig. 2B, D). These effects were further confirmed by 5mC dot blot analysis (Fig. 2E, F). Collectively, these results revealed that long-term aberrant mTOR activation (in HCC patients) was associated with the dysregulation of DNA methylation spectrum, and short-term intervention in mTOR activities (in HCC cell lines) could also further modulate the DNA methylation profile, indicating that crosstalk between mTOR signalling and DNA methylation might exist.

### mTOR signalling regulated DNMT1 expression in HCC

**Fig. 3:**
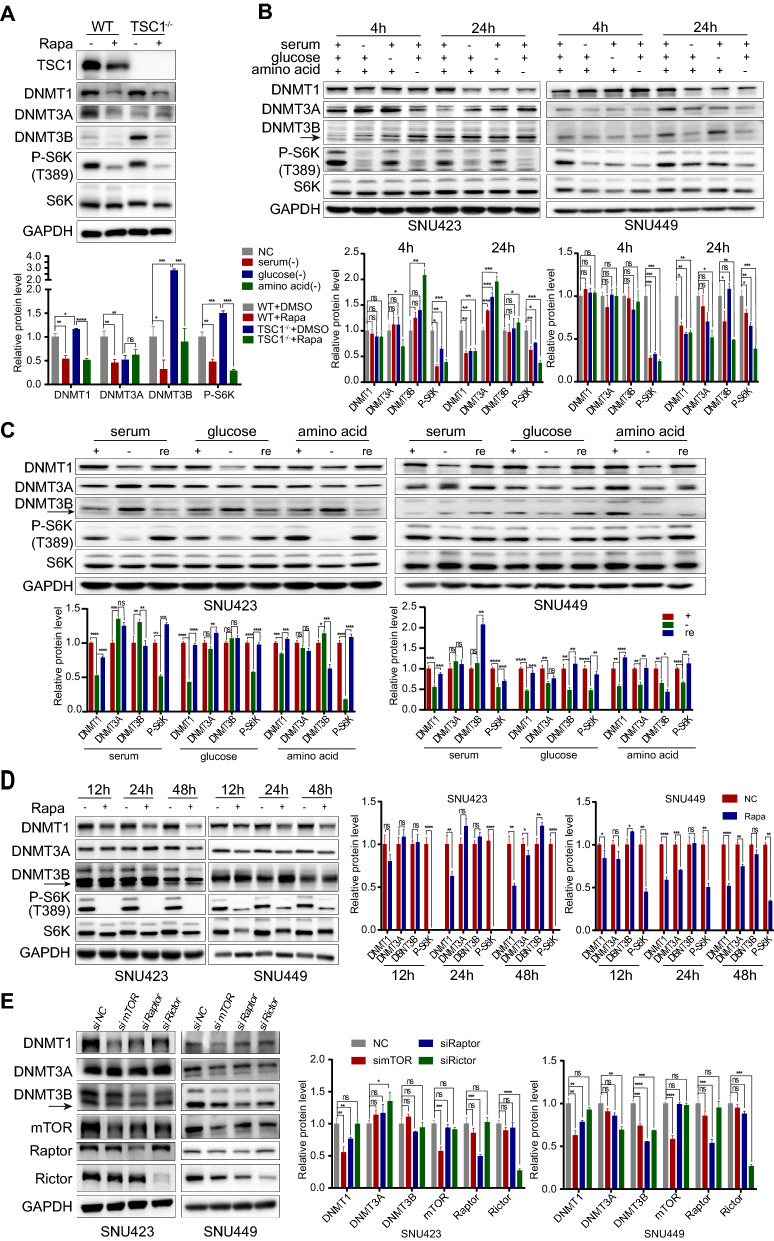
mTOR hyperactivation induces aberrant expression of DNMTs. **A** Protein levels of DNMTs, S6K, P-S6K, and TSC1 determined by Western blot analysis in WT and TSC1^−/−^ MEFs after treatment with DMSO or rapamycin (500 nM) for 24 h. **B** Protein levels of DNMTs, S6K and P-S6K determined by Western blot analysis in SNU423 and SNU449 cells subjected to starvation (cultured in complete medium without serum, glucose, and amino acids) for 4 h or 24 h.** C** Protein levels of DNMTs, S6K and P-S6K determined by Western blot analysis in SNU423 and SNU449 cells starved for 24 h and then recovered by culture in complete medium. **D** Effect of inhibiting mTOR signalling with rapamycin on DNMT and P-S6K protein expression. Protein levels of DNMTs, S6K and P-S6K determined by Western blot analysis in SNU423 and SNU449 cells treated with rapamycin (500 nM) for the indicated periods.** E** Protein levels of DNMTs, mTOR, Raptor, and Rictor determined by Western blot analysis in SNU423 and SNU449 cells after siRNA transfection for 48 h. The solid arrows pointed to the correct location of DNMT3B. The values for P-S6K were normalized against the band intensities of S6K. Data were presented as mean ± SD and each assay was performed for three times. Ns, not significant, *P < 0.05, **P < 0.01, ***P < 0.001, ****P < 0.0001

**Fig. 4:**
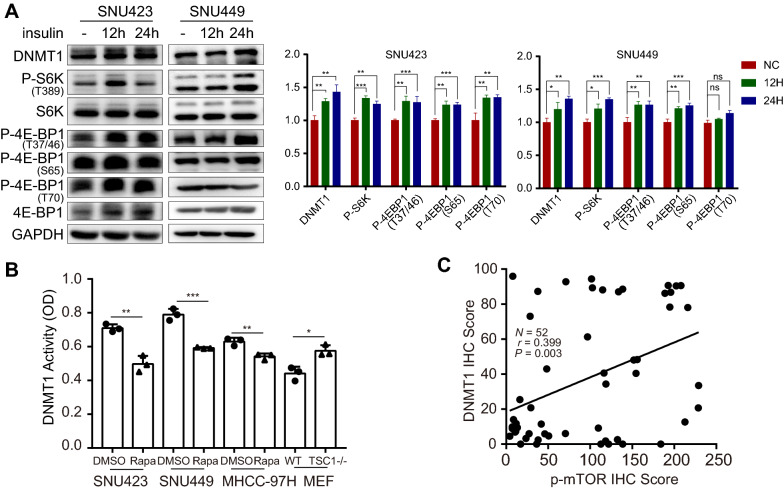
mTOR hyperactivation upregulates the expression and activity of DNMT1. **A** Protein levels of DNMT1, S6K, P-S6K, P-4E-BP1 (T37/46), P-4E-BP1 (S65), P-4E-BP1 (T70), and 4E-BP1 determined by Western blot analysis in SNU423 and SNU449 cells stimulated with 100 ng/ml insulin for 12 h or 24 h. The values for P-S6K and P-4E-BP1 were normalized against the band intensities of S6K and 4E-BP1. **B** DNMT1 activity was detected in SNU423, SNU449 and MHCC-97H cells treated with DMSO and rapamycin (500 nM), as well as in WT and TSC1^−/−^ MEFs, using a DNMT1 assay kit. The *y*-axis indicates DNMT1 activity as represented by the OD value. Data were presented as mean ± SD and each assay was performed for three times. **C** Linear correlation between p-mTOR IHC scores and DNMT1 IHC scores in tumoral regions of HCC patients. Correlations were analysed by Spearman’s rank correlation coefficient test. Ns, not significant, *P < 0.05, **P < 0.01, ***P < 0.001, ****P < 0.0001

To determine which factor mediates the change in the DNA methylation profile upon mTOR activation, we screened the major "writers" (DNA methyltransferases 1, 3A and 3B) of DNA methylation. We found that DNMT1 and DNMT3B expression was upregulated when mTORC1 was activated in TSC1^−/−^ MEFs. In contrast, the mTOR inhibitor rapamycin downregulated DNMT1 and DNMT3B (Fig. 3A). However, these effects were not found for DNMT3A, suggesting that mTORC1 might modify DNA methylation patterns mainly by regulating DNMT1 and DNMT3B. Similar to the effect of rapamycin treatment, nutrient deprivation-mediated mTORC1 suppression markedly decreased DNMT1 expression in both SNU423 and SNU449 cells (Fig. 3B). Additionally, restoring the nutrient status of cells significantly increased the expression level of DNMT1 (Fig. 3C). Treating SNU423 and SNU449 cells with rapamycin also resulted in a significant time-dependent decrease in DNMT1 expression (Fig. 3D). To clarify the relationship between mTOR and DNMTs, we further knocked down mTOR, the core component of mTOR complex as well as the critical components of mTORC1 and mTORC2—i.e., Raptor and Rictor, respectively. The nutrient sensor mTORC1 showed an effect on DNMT1 in both SNU423 and SNU449 cells (Fig. 3E). Collectively, these results showed that DNMT1 expression changed most consistently in tandem with the status of mTORC1, indicating that DNMT1 is most likely the downstream factor of mTORC1 in modifying DNA methylation patterns. Furthermore, adding insulin, which can activate mTORC1, upregulated DNMT1 (Fig. 4A). We also performed a DNMT1 enzymatic activity assay and found that DNMT1 activity was attenuated when mTOR was inhibited. Conversely, mTOR activation enhanced the activity of DNMT1 (Fig. 4B). In addition, IF analysis showed that mTOR signalling mainly influenced the expression level but not the nuclear localization of DNMT1 (Additional file 1: Fig. S2). These observations implied that hyperactivation of mTORC1 induces aberrant DNA methylation patterns, most likely by upregulating the expression and activity of DNMT1. Moreover, we also evaluated the relationship between the mTOR signalling and the DNMT1 level in tumoral regions of HCC patients, and found that the DNMT1 level was positively correlated with the p-mTOR level in HCC tissues (*P* = 0.003, Fig. 4C). The result was consistent with the observations of cell lines.

### mTORC1 upregulated DNMT1 by enhancing its translational efficiency

**Fig. 5:**
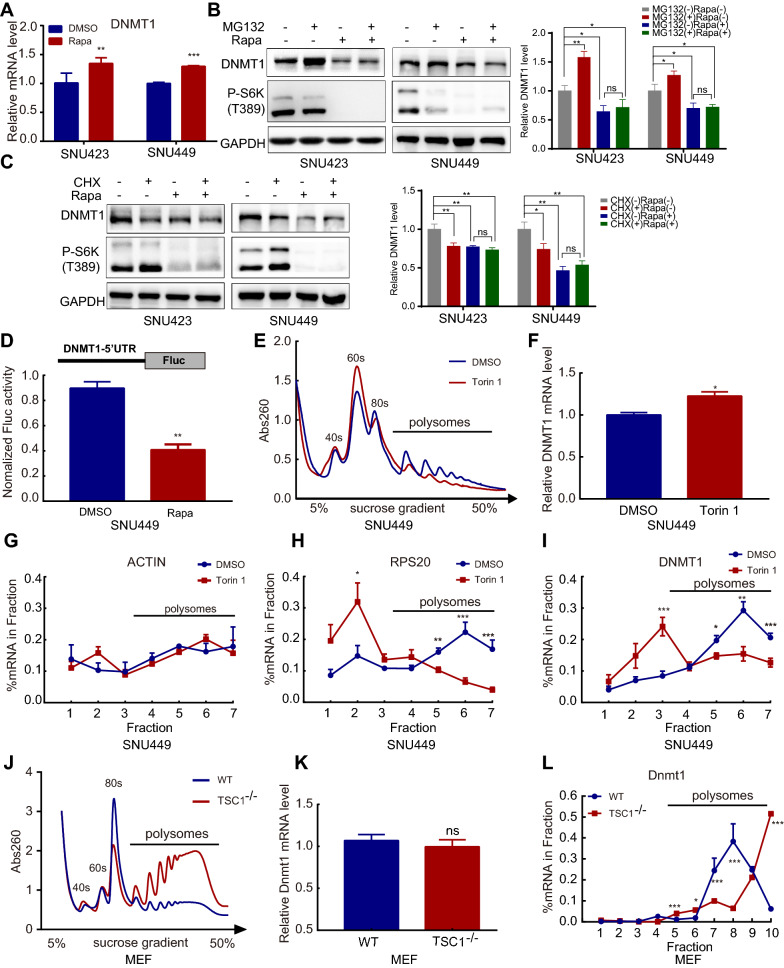
Inhibition of mTOR suppresses the DNMT1 translation process.** A** RT–qPCR analysis of DNMT1 mRNA expression in SNU423 and SNU449 cells treated with rapamycin (500 nM) for 24 h.** B** and** C** Protein levels of DNMT1 and P-S6K determined by Western blot analysis in SNU423 and SNU449 cells treated with or without rapamycin (500 nM) in the presence of MG-132 (20 μM) (**B**) or CHX (50 μM) (**C**) for 12 h. **D** SNU449 cells were transfected with the modified pGL3 plasmid and treated with rapamycin (500 nM) and vehicle (DMSO). mRNA levels were measured by RT–qPCR, and the luciferase activity was normalized to the transcription level. **E** Polysome profiles showing the effect of the mTOR signalling inhibitor Torin1 on global translation in SNU449 cells. SNU449 cells were subjected to nutrient deprivation (maintained in 0.1% FBS) for 16 h and were then incubated with nutrient-replete medium (10% FBS) containing Torin1 (250 nM) for 4 h. DMSO was used as a control.** F** RT–qPCR analysis of mRNA levels in the input lysate from the polysome analysis described in **E**. **G–I** The ACTB, RPS20, and DNMT1 mRNA abundances in the fractions from **E** were quantified by RT–qPCR and calculated as a percentage of the total in all fractions. **J** Polysome profiles showing global translation in MEFs. **K** RT–qPCR analysis of mRNA levels in the input lysate from the polysome analysis described in **J**. **L** The DNMT1 mRNA abundance in the fractions from **J** was quantified by RT–qPCR and calculated as a percentage of the total in all fractions. Data were presented as mean ± SD and each assay was performed for three times. Ns, not significant, *P < 0.05, **P < 0.01, ***P < 0.001, ****P < 0.0001

To explore the mechanism through which mTORC1 regulates the expression of DNMT1, the transcriptional level was first determined. Unexpectedly, the DNMT1 mRNA level was increased upon treatment with the mTOR inhibitor rapamycin (Fig. 5A), in direct contrast to previous findings that mTORC1 upregulated DNMT1 expression, which indicates that mTORC1 posttranscriptionally regulates DNMT1. We subsequently sought to determine whether mTOR regulates the degradation of DNMT1. DNMT1 is reportedly degraded via the ubiquitin–proteasome system [20]. However, we found that the proteasome inhibitor MG-132 could not rescue the DNMT1 protein level when cells were challenged with rapamycin (Fig. 5B). In addition, the results of treatment with the protein synthesis inhibitor cycloheximide (CHX) indicated that rapamycin treatment could not accelerate the degradation of DNMT1 (Fig. 5C, Additional file 1: Fig. S3A). Moreover, inhibition of the autophagic pathway could not reverse the effect of the mTOR inhibitor (Additional file 1: Fig. S3B). These data suggested that mTOR was unlikely to influence the posttranslational degradation of DNMT1.

Therefore, we focused on the role of mTOR in regulating the process of DNMT1 translation. First, we inserted the 5′UTR of DNMT1 into the pGL3 luciferase vector and transfected it into cells. In the absence of changes in mRNA levels, luciferase activity was reduced by treatment with rapamycin (Fig. 5D). This finding indicated that mTOR likely regulates the translational efficiency of DNMT1 in a 5′UTR-dependent manner. Furthermore, we conducted polysome analysis to investigate whether mTOR can directly regulate DNMT1 translation. The ATP-competitive mTOR inhibitor Torin1 markedly decreased mRNA polysome binding, indicating that the global translational efficiency was decreased (Fig. 5E). The level of DNMT1 mRNA transcripts was also measured in total input RNA and was consistent with previous results indicating that it was slightly increased by Torin 1 treatment (Fig. 5F). Next, we further determined the polysome enrichment on specific genes. Compared to that of ACTB (Fig. 5G), which was reported to not be influenced by mTOR, the mRNA of DNMT1 and the well-known mTOR target gene RPS20 showed a similar distribution of ribosomes (Fig. 5H, I). When challenged with an mTOR inhibitor, the percent of DNMT1 mRNA binding in polysomes was significantly decreased, indicating that protein synthesis was inhibited. The same conclusion was obtained with TSC1^−/−^ MEFs, in which mTOR signalling was hyperactivated, protein synthesis was enhanced, and the amount of Dnmt1 mRNA was enriched in polysomes (Fig. 5J–L). Collectively, these findings demonstrated that the DNMT1 translational efficiency is increased when mTOR signalling is aberrantly activated, resulting in protein accumulation.

### Phosphorylation of 4E-BP1 controlled the translational efficiency of DNMT1

**Fig. 6:**
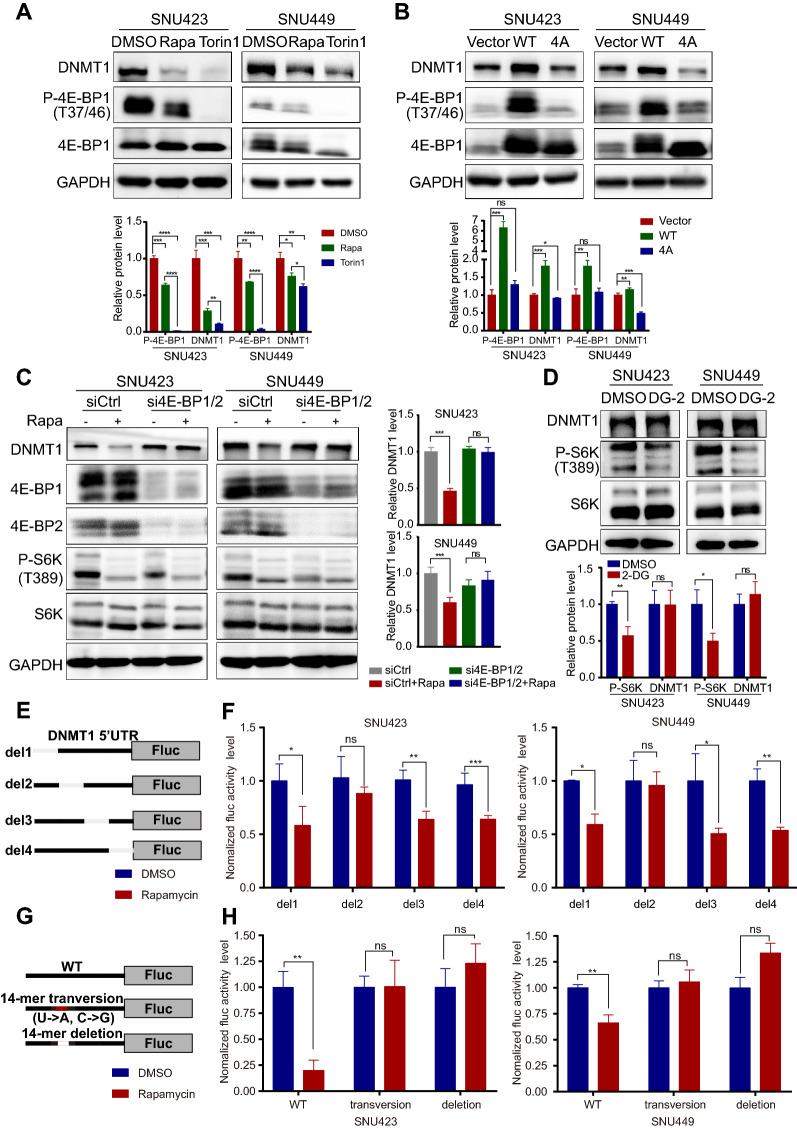
Phosphorylation of 4E-BP1 controls the posttranscriptional expression of DNMT1. **A** Effect of the mTOR inhibitors Torin1 (250 nM) and rapamycin (500 nM) on the DNMT1 level in SNU423 and SNU449 cells. Cells were treated for 24 h with the indicated drugs, and protein levels were subsequently analysed by Western blotting.** B** Protein levels in SNU423 and SNU449 cells transfected with WT 4E-BP1 or the 4E-BP1 mutant (T37A, T46A, S65A, and T70A) (4E-BP1-4A).** C** Representative Western blot analysis of SNU423 and SNU449 cells after 48 h of treatment with 4E-BP1/4E-BP2 siRNA followed by 24 h of treatment with rapamycin (500 nM).** D** Representative Western blot of SNU423 and SNU449 cells after 48 h of DG-2 treatment. The values for P-S6K and P-4E-BP1 were normalized against the band intensities of S6K and 4E-BP1. **E** Schematic showing the insertion of the DNMT1 5′UTR (uc010xlc.2) into pGL3-promoter. **F** Firefly luciferase activity in SNU423 and SNU449 cells after transfection of the respective 5′UTR constructs followed by 24 h of treatment with rapamycin (500 nM). **G** Schematic showing the insertion of the DNMT1 5′UTR constructs [WT, transversion mutant, and PRTE deletion mutant (position − 116 ~ − 129, uc010xlc.2)] into pGL3-promoter. **H** Firefly luciferase activity in SNU423 and SNU449 cells after transfection of the separate 5′UTR constructs followed by 24 h of treatment with rapamycin (500 nM). Data were presented as mean ± SD and each assay was performed for three times. Ns, not significant, *P < 0.05, **P < 0.01, ***P < 0.001, ****P < 0.0001

We then investigated whether the downstream targets of mTORC1, including 4E-BP1 and/or S6 kinase 1/2 (S6K1/2), control the translation of DNMT1. Torin1 efficiently inhibited the phosphorylation of 4E-BP1 and profoundly decreased the DNMT1 level. Rapamycin did not completely block 4E-BP1 phosphorylation, and its inhibitory effect on DNMT1 expression was weaker than that of Torin1 (Fig. 6A). Because 4E-BP1 is a canonical translational repressor that inhibit eIF4E-mediated translation initiation and the consistency between the levels of phosphorylated 4E-BP1 and DNMT1 [21], we hypothesized that DNMT1 might be a downstream effector of 4E-BP1. To validate this hypothesis, a phosphorylation-defective mutant of 4E-BP1 (in which the mTORC1 phosphorylation sites Thr37, Thr46, Ser65 and Thr70 were replaced with Alanine; abbreviated 4E-BP1-4A), with potentially enhanced inhibitory binding activity, showed a suppressive effect on DNMT1 expression compared to WT 4E-BP1 (Fig. 6B). Notably, the knockdown of 4E-BP1 and 4E-BP2 reduced the ability of rapamycin to decrease the expression of DNMT1 (Fig. 6C). However, DG-2, a pharmacological inhibitor of S6K, had no effect on regulating DNMT1 expression (Fig. 6D). These findings indicated that 4E-BP1 is the main downstream effector of oncogenic mTOR that regulates the translational efficiency of DNMT1. However, the 5′UTR of DNMT1 is not a typical terminal oligopyrimidine (TOP) or TOP-like sequence, and genes whose 5′UTR contains such a sequence show more sensitivity to mTOR inhibition [22]. Studies have further shown that pyrimidine-rich translational element (PRTE)-containing 5′UTRs are also sensitive to mTOR regulation [17]. Indeed, we identified several PRTEs in the 5′UTR of DNMT1. Thus, we constructed four 5′UTR deletion mutants of DNMT1 to identify the exact position that eIF4E can recognize (Fig. 6E). Eventually, we identified a potential PRTE whose deletion abolished the mTOR inhibitory effect on DNMT1 (Fig. 6F). To determine the functional role of this PRTE in regulating translational efficiency, we mutated or depleted this PRTE in the 5′UTR of DNMT1 (Fig. 6G), which rendered the 5′UTR of DNMT1 insensitive to inhibition by rapamycin (Fig. 6H). These findings revealed that mTOR regulated the translational efficiency of DNMT1 mainly by regulating the phosphorylation of 4E-BP1 and the subsequent binding of eIF4E to the PRTE sequence in the 5′UTR of DNMT1.

### Combination treatment with mTOR inhibitor and demethylating agent inhibited the growth of liver cancer cells in vitro and in vivo

In the experiments mentioned above, we found that mTOR activation can increase the translational efficiency of DNMT1 and change the genomic methylation spectrum, by which likely to affect tumour progression and the chemosensitivity of cells. By IHC scoring, we further found that both of p-mTOR and DNMT1 levels were positively correlated with the Ki67 levels (a marker of cellular proliferation) in tumoral regions (*P* = 0.002 and *P* < 0.001, Additional file 1: Fig. S4A, B). Moreover, Kaplan–Meier survival analysis revealed a negative correlation between the intratumor DNMT1 level and overall survival (OS, *P* < 0.001; Additional file 1: Fig. S4C) as well as recurrence-free survival (RFS, *P* < 0.001; Additional file 1: Fig. S4E). Patients in the DNMT1^high^ and p-mTOR^high^ groups exhibited the worst OS (5-year OS rate: 17.6%) and RFS (5-year RFS rate: 0%) compared with those in the p-mTOR^high^ and DNMT1^low^ group (5-year OS rate: 44.4%; 5-year RFS rate: 11.1%), the p-mTOR^low^ and DNMT1^high^ group (5-year OS rate: 44.4%; 5-year RFS rate: 14.3%) and the p-mTOR^low^ and DNMT1^low^ group (5-year OS rate: 88.2%; 5-year RFS rate: 69.7%; Additional file 1: Fig. S4D, F). These data in HCC tissues suggested mTOR signalling pathway and DNMT1 might affect cell proliferation and the combination of DNMT1 and p-mTOR levels could represent a more powerful criterion for predicting HCC patients’ survival.

To further sustain the above finding that mTOR signalling pathway might affect cell proliferation and DNMT1 expression, we used various HCC cell lines in vitro. In HCC cell lines responsive to the mTOR inhibitor rapamycin, as the concentration of rapamycin increased, DNMT1 expression was decreased and cell proliferation was inhibited at the same time (Additional file 1: Fig. S5A, B). In addition, it seems that DNMT1 level was positively correlated with cell proliferation when challenging with rapamycin (Additional file 1: Fig. S5C). Taken together, these results collectively demonstrated that mTOR affected cell proliferation in part by regulating DNMT1.

**Fig. 7:**
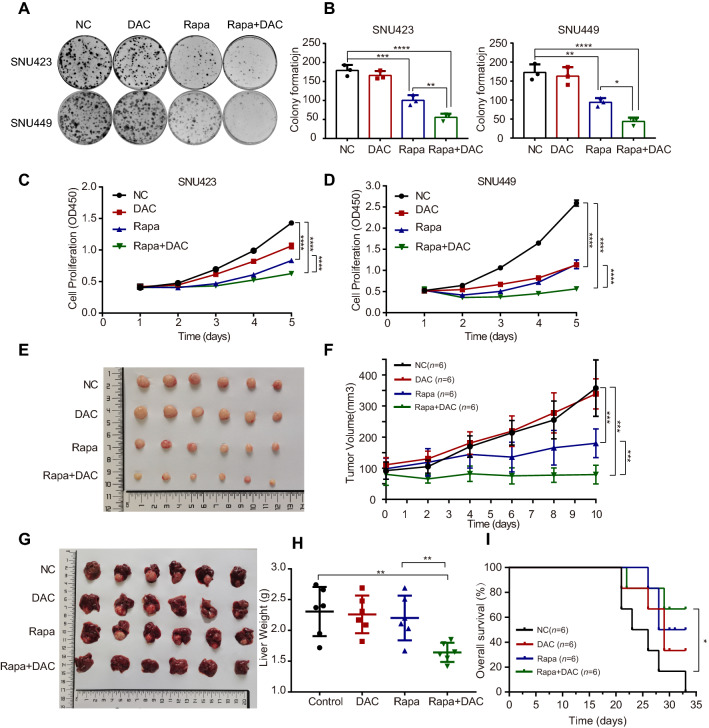
The combination of rapamycin and decitabine inhibits the growth of liver cancer cells in vitro and in vivo*.* **A** A colony formation assay was performed with SNU423 and SNU449 cells treated with DMSO or decitabine (DAC) or rapamycin (Rapa) alone or in combination. **B** Quantification of colony formation in **A**. **C**, **D** Cell proliferation was measured by a CCK8 assay. SNU423 and SNU449 cells were treated with DMSO or decitabine or rapamycin alone or in combination for different times. Data were presented as mean ± SD and each assay was performed for three times. **E** The combination of decitabine and rapamycin significantly suppressed subcutaneous tumour growth in mice. **F** Tumour volume was measured daily. The data (mean ± SD; n = 6) were analysed by repeated-measures ANOVA; ***P < 0.001. **G** The combination of decitabine and rapamycin significantly suppressed the growth of orthotopic xenograft tumours in the liver. **H** The liver weight was decreased in the drug combination group in the in situ liver tumour model (unpaired t test). **I** The combination of decitabine and rapamycin significantly improved the overall survival in mice with orthotopic liver tumors. Overall survival was estimated using the Kaplan–Meier method and compared using the log‒rank test. *P < 0.05, **P < 0.01, ***P < 0.001, ****P < 0.0001

To determine whether mTOR-mediated regulation of DNA methylation can enhance the therapeutic effect of canonical epigenetic drugs, we assessed the combinatorial effect of decitabine, an FDA-approved DNA demethylating agent, with the mTOR inhibitor rapamycin. The combination showed a markedly synergistic effect on inhibiting colony formation (Fig. 7A, B) and cell proliferation (Fig. 7C, D) in two HCC cell lines. In addition, we evaluated the in vivo antitumour activity of the combination of rapamycin with decitabine in subcutaneous transplanted tumour models. Although rapamycin alone had statistically significant antitumour activity, the combination of decitabine and rapamycin showed a more potent effect (Fig. 7E, F). This combination therapy also showed a superior inhibitory effect on liver tumours in situ. However, rapamycin monotherapy had no evident effect (Fig. 7G, H). Moreover, we also performed long-term survival analysis in mice model of orthotopic tumor and showed that combination therapy significantly improved the overall survival compared with control (*P* = 0.033, Fig. 7I). IHC was also performed to measure p-mTOR, DNMT1, 5mC and Ki67 in the mouse sections of each group, and the combination of rapamycin and decitabine showed a synergistic effect to reduce their signals (Additional file 1: Fig. S6A, B). Negative correlations were detected between overall survival and the intratumor p-mTOR level (*P* = 0.035), 5mC level (*P* = 0.016) and DNMT1 level (*P* < 0.001, Additional file 1: Fig. S6C). These findings provide an experimental basis for combination treatment with mTOR inhibitors and DNA demethylating agents for liver cancer therapy.

## Discussion

Although aberrant DNA methylation profiles have been widely involved in multiple stages of HCC, including cancer initiation, progression and metastasis [23], the mechanism through which DNA methylation profiles are disrupted is not well understood. In this study, we found a novel role of mTOR in the induction of DNA methylation in HCC. The DNA methylation level was positively associated with activation of mTOR signalling in both HCC tissues and cell lines. In line with our findings, Naneel et al. [24] showed that the activation of mTOR signalling can modulate DNA methylation by affecting serine/1C metabolism in primary pancreatic ductal epithelial cells of mice. In addition, patients in the p-mTOR^high^ and 5mC^high^ group exhibited the worst prognosis. These data further indicated that mTOR signalling and DNA methylation might synergistically potentiate tumour progression in HCC.

Moreover, we showed that abnormal activation of mTORC1 regulated the translational efficiency of DNMT1, which can modify global DNA methylation profiles. In the short-term model, we selectively inhibited the mTOR pathway by utilizing rapamycin or Torin1 and found that mTOR suppression significantly downregulated DNMT1 expression and led to a slight decrease in global DNA methylation. Oncogenic pathways have been reported to be able to hijack sensing mechanisms to sustain one-carbon metabolism, which provides critical metabolites to support the rapid proliferation of cancer cells [25]. Collectively, the results demonstrate that altered DNA methylation patterns along with this metabolic reprogramming likely generate conditions suitable for tumour growth. Notably, in our model, in which cancer cells were treated with mTOR inhibitors, these adapted DNA methylation profiles underwent further changes, which may be partially connected to the antitumour effects of mTOR inhibitors and explain why drug resistance develops rapidly in some cases. Identifying specific DNA methylation loci sensitive to mTOR inhibition may improve our ability to predict the therapeutic effect of targeting mTOR.

The establishment of DNA methylation profiles is commonly believed to be mediated by the de novo methyltransferases DNMT3A and DNMT3B, whereas DNMT1 ensures the maintenance of methylation patterns during replication [26]. However, emerging data have also revealed an overlapping function between these two kinds of methyltransferases [20]. Our data showed that DNMT1 was the most sensitive to mTOR pathway modulation; however, DNMT3A and DNMT3B were also influenced by mTOR activity. We could not exclude the possibility that DNMT3A and DNMT3B play a role in mTOR-mediated regulation of DNA methylation profiles. To determine the mechanism through which mTOR regulates DNMT expression, we focused on the most mTOR-sensitive, DNMT1. mTOR can specifically regulate the translational efficiency of a subset of mRNAs with 5′ TOP or TOP-like motifs in their 5′UTRs in a 4E-BP1-dependent manner [22]. Although we did not find a typical TOP motif in the 5′UTR of DNMT1, we did identify some PRTEs that also showed high sensitivity to mTOR regulation [17]. As expected, deletion or mutation of this PRTE-containing fragment impaired the regulation of DNMT1 by mTOR. Thus, we discovered that the oncogenic mTOR signalling pathway regulates DNA methylation by translationally regulating critical methyltransferases. It was reported that DNMT1-mediated PTEN hypermethylation causes loss of PTEN expression followed by activation of PI3K/AKT [27]. However, we did not observe a marked change when DNMT1 was knocked down or inhibited (Additional file 1: Fig. S7). Thus, the interaction of mTOR pathway activity with the methylation level is worth exploring.

Drug combination is a promising strategy to enhance therapeutic efficacy while minimizing toxicity. Because the activation of mTOR signalling contributes to aberrant DNA methylation, inhibitors of mTOR signalling and DNMTs can rationally be combined to target the expression and activity of DNMTs concurrently. We found that rapamycin (a selective mTOR inhibitor) synergized with decitabine (DNMT inhibitor) to inhibit HCC in vitro and in vivo. Altogether, these data suggest that targeting the mTOR-DNMT axis might represent a novel therapeutic approach in HCC.

## Conclusions

**Fig. 8:**
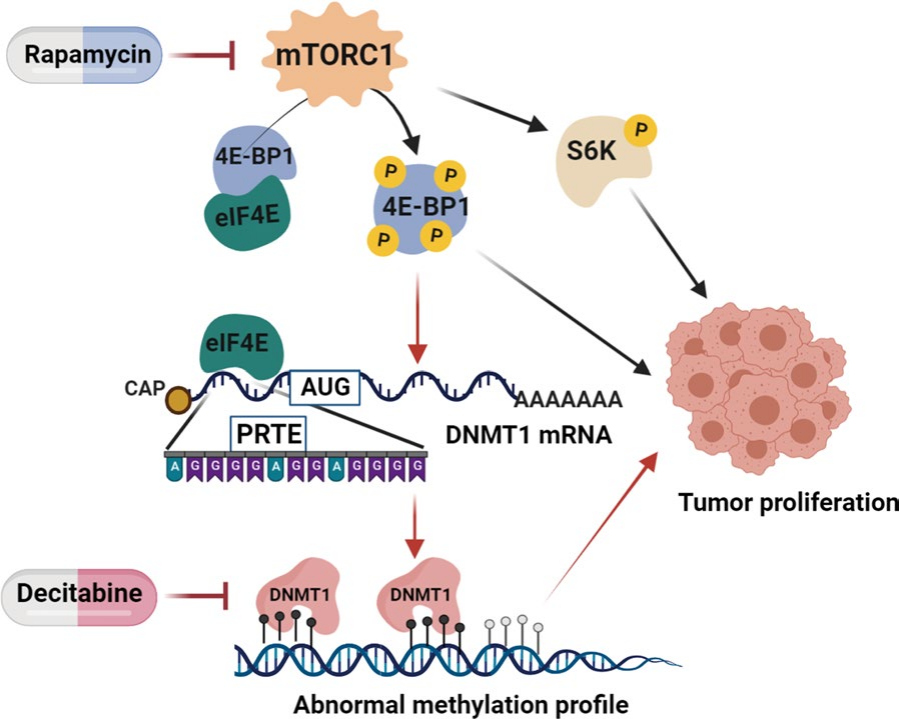
Schematic diagram of the mechanism through which mTOR signalling regulates DNA methylation in HCC. Abnormally activated mTOR increases the phosphorylation of 4E-BP1, enhancing eIF4E release from the 4E-BP1/eIF4E complex. Free eIF4E can bind to the 5′UTR of DNMT1, which contains a PRTE, enhancing the translational efficiency of DNMT1. This process can regulate the DNA methylation profile during carcinogenesis, possibly promoting the development of HCC. Combination treatment with mTOR and epigenetic inhibitors synergistically inhibited HCC

In summary, we discovered that mTOR regulates DNMT1 in a 4E-BP1-dependent manner to affect DNA methylation profiles, which emphasizes that dysregulation of oncogenic pathways can alter the epigenetic status to affect tumour growth (Fig. 8). Given that both mTOR inhibitors and DNA methylation inhibitors are widely used in cancer therapy, we believe that determining the exact crosstalk between the mTOR pathway and DNA methylation can improve our ability to predict drug responses and develop better therapeutic regimens.

## Supplementary Information

**Additional file 1: Figure S1**. The association of the mTOR signalling pathway and DNA methylation in adjacent normal tissues. **Figure S2**. Nutritive elements influence DNMT1 protein level but not its distribution. **Figure S3**. The degradation process of DNMT1 is not repressed by inhibition of mTOR. **Figure S4**. The p-mTOR level and DNMT1 level are positively correlated with cell proliferation and tumor progression in HCC patients. **Figure S5**. DNMT1 levels are positively correlated with cell proliferation in rapamycin-treated HCC cell lines. **Figure S6**. The combination of rapamycin and decitabine improves the long-term survival in mice with orthotopic liver tumors. **Figure S7**. Inhibition of DNMT1 has little effect on Akt-mTOR signaling pathway. **Table S1**. Clinical Features of 52 HCC Patients. **Table S2**. Primers and siRNAs used in the study. **Table S3**. List of Antibodies used in Western blot.

## Data Availability

These data has not been previously reported and is not under consideration for publication elsewhere. All the raw data are available from the corresponding author on reasonable request.

## Abbreviations

HCC: Hepatocellular carcinoma
mTOR: Mammalian/mechanistic target of rapamycin
5mC: 5-Methylcytosine
DNMT1: DNA methyltransferase 1
OS: Overall survival
RFS: Recurrence-free survival
PRTE: Pyrimidine rich translational element
eIF4E: Eukaryotic translation initiation factor 4E
4E-BP1: EIF4E binding protein 1
IHC: Immunohistochemistry
IF: Immunofluorescence
