# Supplementary material for: The activation of mTOR signalling modulates DNA methylation by enhancing DNMT1 translation in hepatocellular carcinoma
Source: J Transl Med. 2023 Apr 23;21:276. doi: 10.1186/s12967-023-04103-9 (PMC10124003; doi:10.1186/s12967-023-04103-9)
Supplement: Supplementary file 1 — Additional file 1: Figure S1. The association of the mTOR signalling pathway and DNA methylation in adjacent normal tissues. Figure S2. Nutritive elements influence DNMT1 protein level but not its distribution. Figure S3. The degradation process of DNMT1 is not repressed by inhibition of mTOR. Figure S4. The p-mTOR level and DNMT1 level are positively correlated with cell proliferation and tumor progression in HCC patients. Figure S5. DNMT1 levels are positively correlated with cell proliferation in rapamycin-treated HCC cell lines. Figure S6. The combination of rapamycin and decitabine improves the long-term survival in mice with orthotopic liver tumors. Figure S7. Inhibition of DNMT1 has little effect on Akt-mTOR signaling pathway. Table S1. Clinical Features of 52 HCC Patients. Table S2. Primers and siRNAs used in the study. Table S3. List of Antibodies used in Western blot. [file 12967_2023_4103_MOESM1_ESM.docx]

**Additional file 1**

**The activation of mTOR signalling modulates DNA methylation by enhancing** **DNMT1 translation in** **hepatocellular carcinoma**

Mengke Chen^1,2†^, Yi Fang^2†^, Meinong Liang^2†^, Ning Zhang^3^, Xinyue Zhang^1,2^, Lixia Xu^1^, Xuxin Ren^1,2^, Qingfeng Zhang^4^, Yufeng Zhou^4^, Sui Peng^2^, Jun Yu^2^, Judeng Zeng^5*^, Xiaoxing Li^1,2*^

^1^ Department of Oncology, Sun Yat-sen University First Affiliated Hospital, Guangzhou, China.

^2^ Institute of Precision Medicine, Sun Yat-sen University First Affiliated Hospital, Guangzhou, China.

^3^ Department of Gastroenterology, Sun Yat-sen University First Affiliated Hospital, Guangzhou, China.

^4^ Sun Yat-Sen University Cancer Center, Guangzhou, China.

^5^ Department of Anaesthesia and Intensive Care, The Chinese University of Hong Kong, Hong Kong, China.

^*^Correspondence: Xiaoxing Li, [lixiaox23@mail.sysu.edu.cn](mailto:lixiaox23@mail.sysu.edu.cn); Judeng Zeng, [zjdseed@163.com](mailto:zjdseed@163.com)

^†^ Mengke Chen, Yi Fang and Meinong Liang contributed equally to this work

**
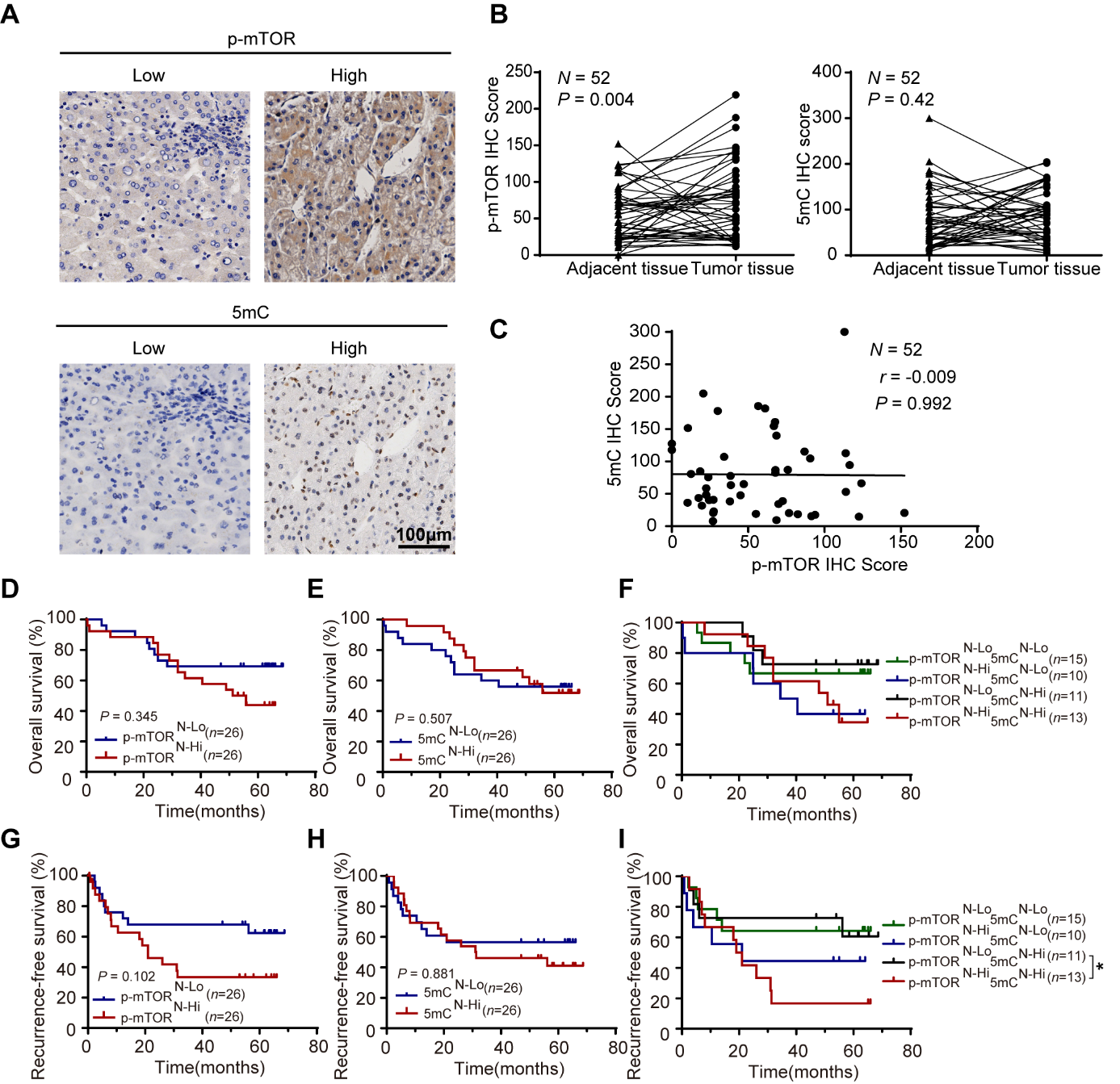
**

**Fig. S1 The association of the mTOR signalling pathway and DNA methylation in adjacent normal tissues**

**A** Representative IHC images demonstrate low (left) and high (right) scores for p-mTOR (above) and 5mC (under). Scale bar = 100 μm. **B** Scatter plots of p-mTOR (left) and 5mC (right) IHC scores in paired HCC and adjacent noncancerous liver tissues (paired t-test). **C** Linear correlation between p-mTOR IHC scores and 5mC IHC scores in adjacent normal tissues. Correlations were analysed by Spearman’s rank correlation coefficient test. **D-F** Cumulative overall survival curves of HCC patients based on p-mTOR and 5mC in adjacent normal tissues. **G-I** Recurrence-free survival curves of HCC patients based on p-mTOR and 5mC in adjacent normal tissues. The patients were divided into two groups according to the median IHC score of p-mTOR and 5mC. N, adjacent normal region; lo, low; hi, high. **P* < 0.05, ***P* < 0.01, ****P* < 0.001.


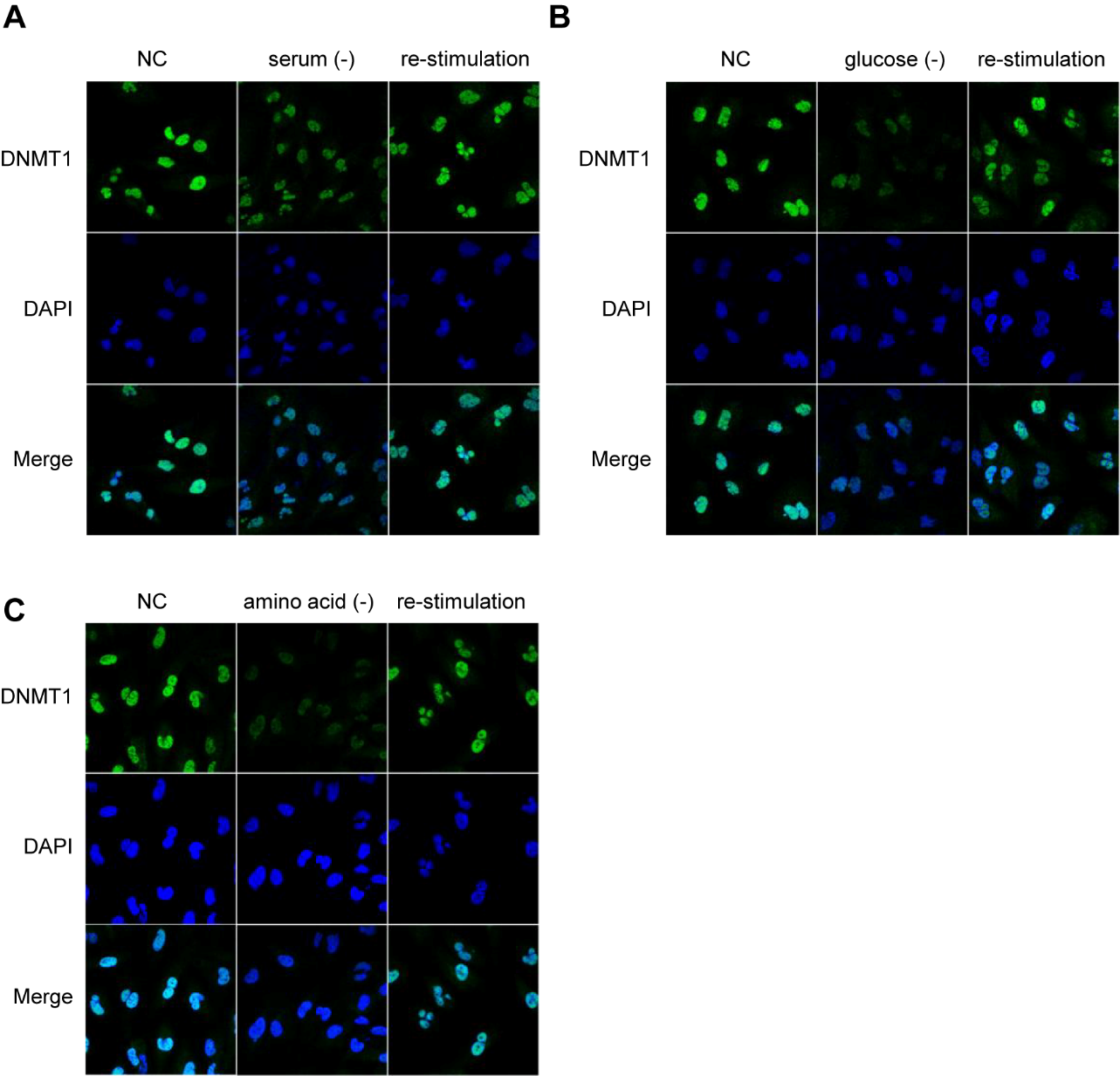


**Fig. S2 Nutritive elements influence DNMT1 protein level but not its distribution**

**A-C** Representative IF staining of DNMT1 in SNU423 cells, and nuclei were counterstained by DAPI. Compared to control group, in which cells were cultured in complete medium, cells of experiment were starved (complete medium in absence of serum (**A**), glucose (**B**), and amino acid (**C**) for 24h and then recovered with complete medium for 24h.


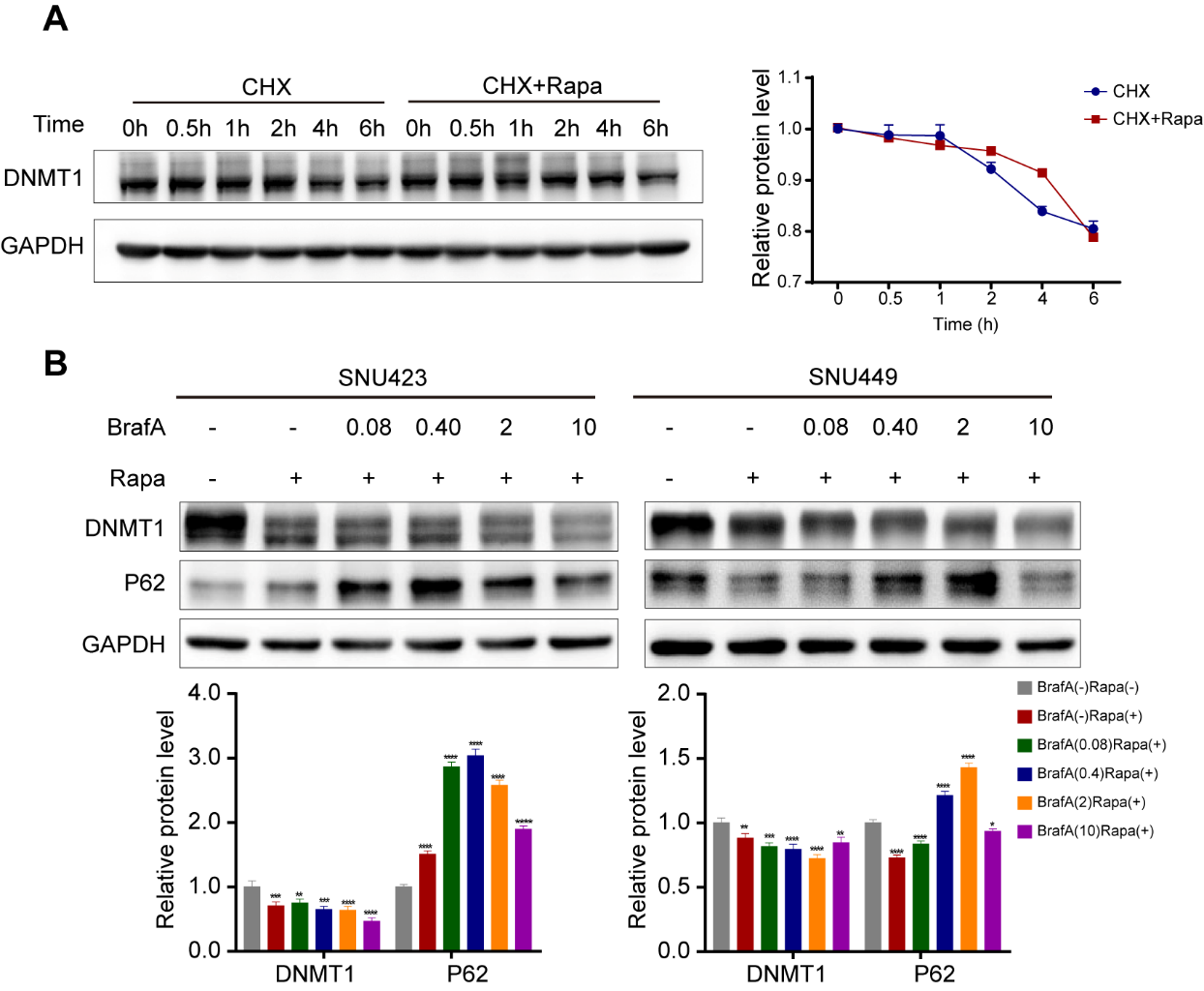


**Fig. S3 The degradation process of DNMT1 is not repressed by inhibition of mTOR**

**A** Protein levels of DNMT1 by Western Blot in SNU449 cells that treated with or without rapamycin (500 nM) in the presence of CHX (50 μM) for the indicated periods. The ratio of DNMT1/GAPDH was used to calculate DNMT1 stability. The *x*-axis indicated time while the *y*-axis indicated the ratio of DNMT1/GAPDH. **B** Protein levels of DNMT1 and P62 detected by Western Blot in SNU423 and SNU449 cells that treated with or without rapamycin (500 nM) in the presence of autophagy inhibitor bafilomycin A1 of different concentration for 12h. The increased P62 level hint the inhibition of autophagy pathway. Data were presented as mean ± SD and each assay was performed for three times. **P* < 0.05, ***P* < 0.01, ****P* < 0.001, *****P* < 0.0001, compared to control group.


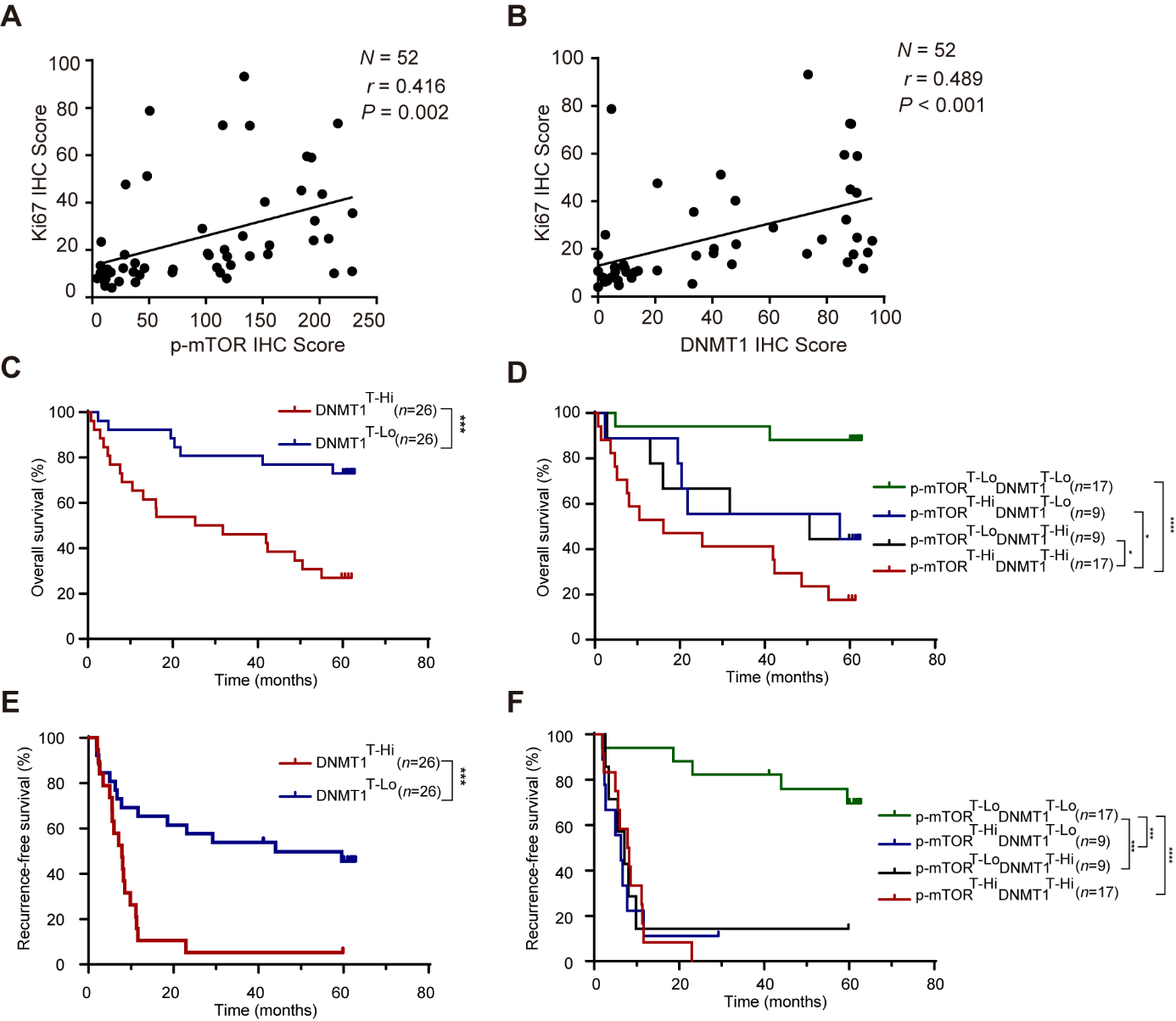


**Fig. S4 The p-mTOR level and DNMT1 level are positively correlated with cell proliferation and tumor progression in HCC patients**

**A** Linear correlation between p-mTOR IHC scores and Ki67 IHC scores of tumoral regions. **B** Linear correlation between DNMT1 IHC scores and Ki67 IHC scores of tumoral regions. Correlations were analysed by Spearman’s rank correlation coefficient test. **C** Cumulative overall survival curves of HCC patients based on tumoral DNMT1 levels. **D** Cumulative overall survival curves of HCC patients based on combination of tumoral DNMT1 and p-mTOR levels. **E** Recurrence-free survival curves of HCC patients based on tumoral DNMT1 levels. **F** Recurrence-free survival curves of HCC patients based on combination of tumoral DNMT1 and p-mTOR levels. **P* < 0.05, ***P* < 0.01, ****P* < 0.001, *****P* < 0.0001.


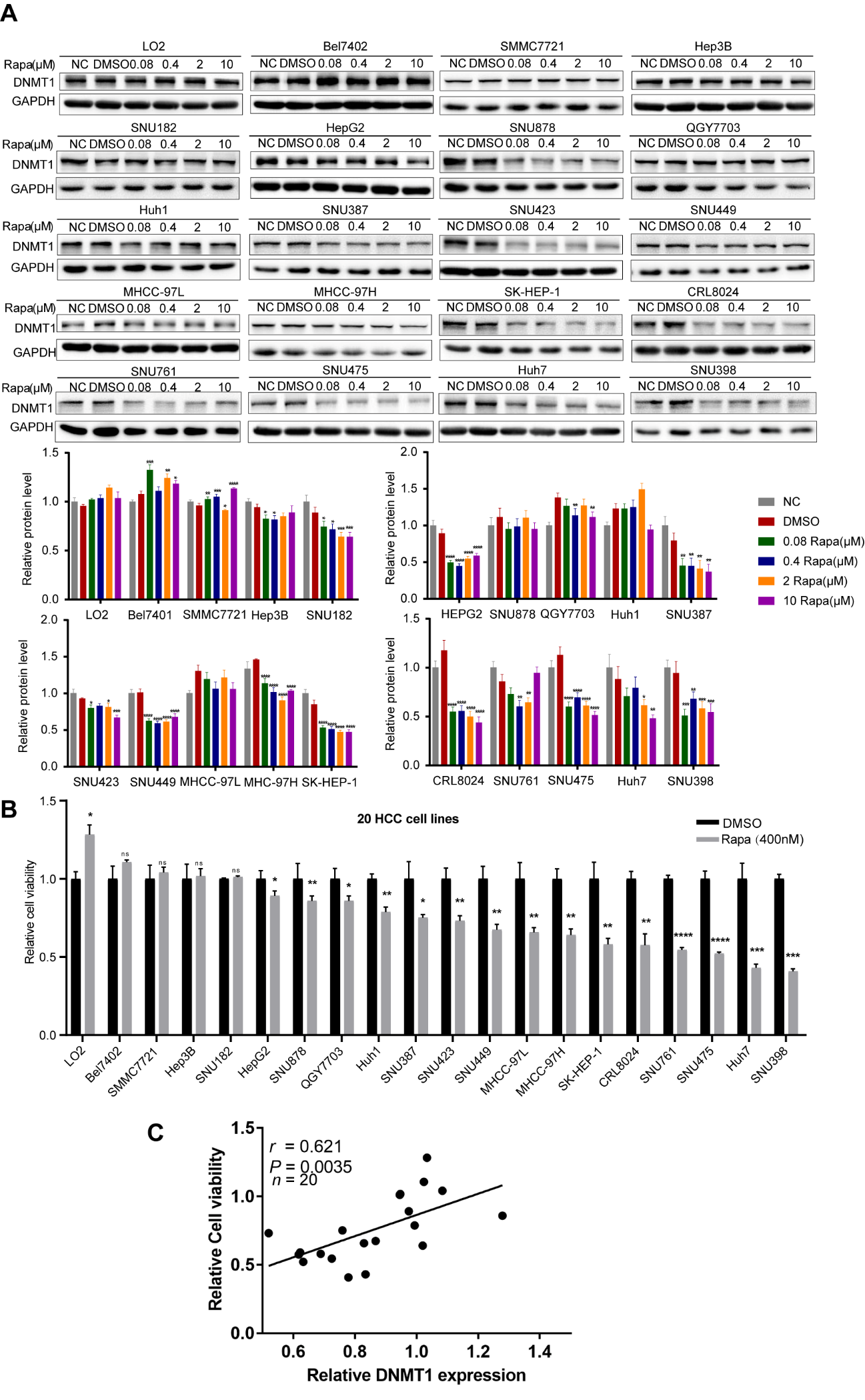


**Fig. S5 DNMT1 levels are positively correlated with cell proliferation in rapamycin-treated HCC cell lines**

**A** Protein levels of DNMT1 determined by Western blot analysis in LO2, Bel7402, SMMC7721, Hep3B, SNU182, HepG2, SNU878, QGY7703, Huh1, SNU387, SNU423, SNU449, MHCC-97L, MHCC-97H, SK-HEP-1, CRL8024, SNU761, SNU475, Huh7 and SNU398 cells after treatment with normal control, DMSO or rapamycin (0.08, 0.4, 2 and 10 μM) for 24 h. **B** Cell proliferation was measured by a CCK8 assay. The HCC cell lines were treated with DMSO or 0.4 μM rapamycin for 24 h. **C** Linear correlation between the relative DNMT1 expression and the relative cell viability in HCC cell lines. The relative DNMT1 expression referred to the DNMT1expression in HCC cells treated by rapamycin divided by the DNMT1 expression in HCC cells treated by DMSO for 24 h. The relative cell viability meant that cell viability in HCC cells treated by rapamycin at 24 h divided by the cell viability in HCC cells before treatment at 0 h. Correlations were analyzed by Spearman’s rank correlation coefficient test. Data were presented as mean ± SD and each assay was performed for three times. **P* < 0.05, ***P* < 0.01, ****P* < 0.001, *****P* < 0.0001, compared to DMSO group.

**
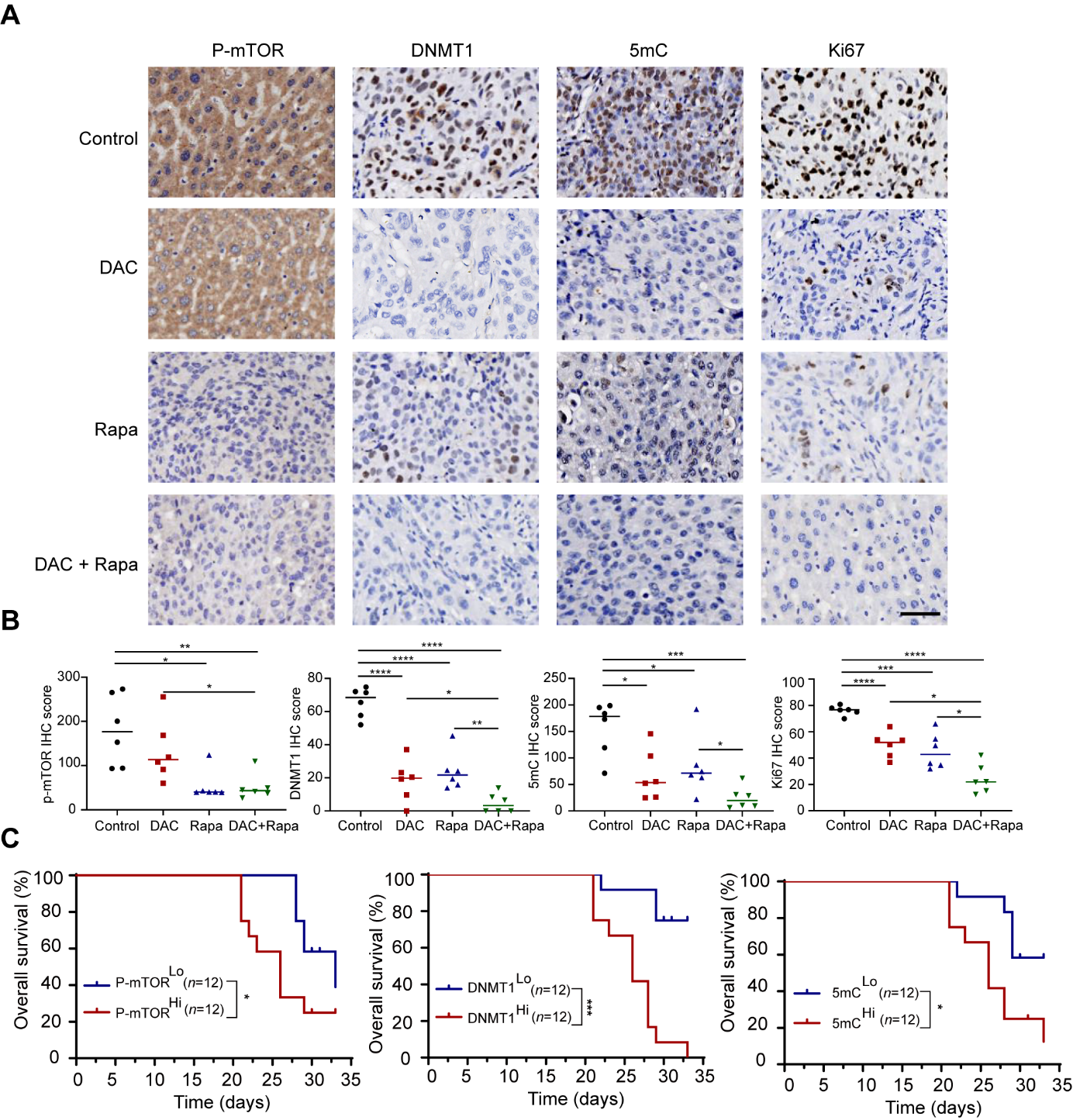
**

**Fig. S6 The combination of rapamycin and decitabine improves the long-term survival in mice with** **orthotopic liver tumors**

**A, B** Representative IHC images (**A**) and statistics analysis (**B**) of p-mTOR, DNMT1, 5mC and ki67 in groups of control, rapamycin, decitabine and combination treatment. Scale bar = 100 μm. **C** Cumulative overall survival curves based on p-mTOR (left), DNMT1 (middle) and 5mC (right). The mice were divided into two groups according to the median IHC score of p-mTOR, DNMT1 and 5mC. **P* < 0.05, ***P* < 0.01, ****P* < 0.001, *****P* < 0.0001.


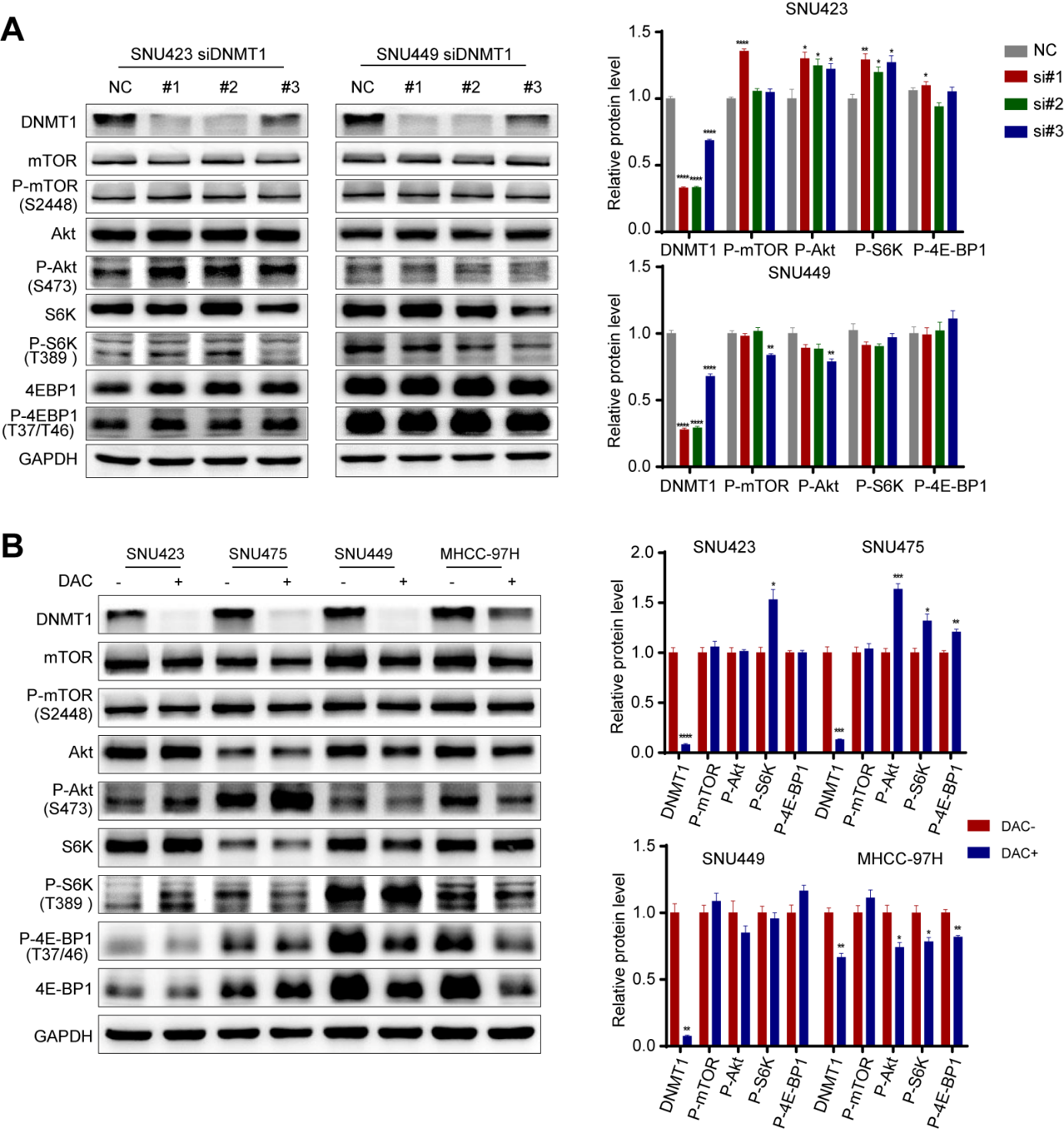


**Fig. S7 Inhibition of DNMT1 has little effect on Akt-mTOR signaling pathway**

**A** Protein levels of DNMT1, mTOR, P-mTOR (S2448), Akt, P-Akt (S473), S6K, P-S6K (T389), 4E-BP1, P-4E-BP1 (T37/T46) detected by Western Blot in SNU423 and SNU449 cells after knocking down DNMT1 for 48h. **B** Protein levels of DNMT1, mTOR, P-mTOR (S2448), Akt, P-Akt (S473), S6K, P-S6K (T389), 4E-BP1, P-4E-BP1 (T37/T46) detected by Western Blot in SNU423, SNU475, SNU449 and MHCC-97H cells that were treated with Decitabine (10 μM) for 72h. The values for P-mTOR, P-Akt, P-S6K and P-4E-BP1 were normalized against the band intensities of mTOR, Akt, S6K and 4E-BP1. Data were presented as mean ± SD and each assay was performed for three times. **P* < 0.05, ***P* < 0.01, ****P* < 0.001, *****P* < 0.0001, compared to control group.

**Table S1. Clinical Features of 52 HCC Patients**

| **Variables** | **Cases** |  | **Variables** | **Cases** |  |  |
| --- | --- | --- | --- | --- | --- | --- |
| Gender |  |  | Tumor number |  |  | |
| Male | 42 |  | 1 | 44 |  | |
| Female | 10 |  | >1 | 8 |  | |
| Age-years |  |  | Tumor size (cm) |  |  |  |
| ≤60 | 37 |  | ≤5 | 25 |  |  |
| >60 | 15 |  | >5 | 27 |  |  |
| HBV |  |  | Tumor grade |  |  |  |
| +^a^ | 39 |  | I-II | 9 |  |  |
| -^a^ | 12 |  | >II | 10 |  |  |
| No data | 1 |  | No data | 33 |  |  |
| HCV |  |  | Tumor capsule |  |  |  |
| +^a^ | 1 |  | None/incomplete | 8 |  |  |
| -^a^ | 50 |  | Complete | 10 |  |  |
| No data | 1 |  | No data | 34 |  |  |
| AFP (ng/ml) |  |  | BCLC stage |  |  |  |
| ≤400 | 23 |  | 0/A | 18 |  |  |
| >400 | 28 |  | B/C/D | 15 |  |  |
| No data | 1 |  | No data | 19 |  |  |

^a^+, presence; -, absence.

^b^AJCC, American Joint Committee on Cancer.

**Table S2. Primers and siRNAs used in the study:**

| Name | Sequences (5’-3’) |
| --- | --- |
| DNMT1-F | GAATCAGTTATGTGACTTGGAAACC |
| DNMT1-R | CTAGACGTCCATTCACTTCCC |
| DNMT3A-F | GGACAAGAATGCCACCAAAG |
| DNMT3A-R | CACCAAGACACAATGCGG |
| DNMT3B-F | GCAACCATGTGGACGAGTC |
| DNMT3B-R | GTCTGTGAGGTCGATGGTAAG |
| Firefly-F | GAGGCGAACTGTGTGTGAGA |
| Firefly-R | GGGTGTTGGAGCAAGATGGA |
| Renilla-F | ATAACTGGTCCGCAGTGGTG |
| Renilla-R | TAAGAAGAGGCCGCGTTACC |
| GAPDH-F | CTCCTCCTGTTCGACAGTCAGC |
| GAPDH-R | CCCAATACGACCAAATCCGTT |
| ACTB-F | AGCCTCGCCTTTGCCGA |
| ACTB-R | GCGCGGCGATATCATCATC |
| firefly luciferase-F | ATCCGGAAGCGACCAACGCC |
| firefly luciferase-R | GTCGGGAAGACCTGCCACGC |
| AJAP1-F | GTTAGCACAACGGAGCCTTC |
| AJAP1-R | GATGATCTGATGGACAGCCA |
| B3GALT4-F | CTACCGCAACCTCACCCTAAA |
| B3GALT4-R | AGGGACGTTGACATACACATCA |
| CELSR1-F | CGCTTCCACTTCACCATCTCCCT |
| CELSR1-R | GCCACGGTCGTTGTTGTCTCG |
| CPNE7 -F | GACCTCTTCAGCAAGTCCGAC |
| CPNE7 -R | CACACCGGGTTCAGGTTGT |
| CYS1-F | AGCATCTCAGGAACCAACGG |
| CYS1-R | AACAGCTGGTCAAAGGCACT |
| DLEC1-F | CCAAAACGCGGAGGTCTTTAG |
| DLEC1-R | GGGAGGAATACAAGGAGGACT |
| FBXL16-F | TCATTGACAACTATGCGCTCTC |
| FBXL16-R | GATGCAGTCACTCACGCTCA |
| FOXD1-F | TGAGCACTGAGATGTCCGATG |
| FOXD1-R | CACCACGTCGATGTCTGTTTC |
| LHX2-F | ATGCTGTTCCACAGTCTGTCG |
| LHX2-R | GCATGGTCGTCTCGGTGTC |
| LPAR2-F | CCTGGTCAAGACTGTTGTCATC |
| LPAR2-R | GACTCACAGCCTAAACCATCC |
| MAP6-F | TTGATCGCAGAAGAATACGCAG |
| MAP6-R | GCCTTCCTCGTGGGCTTAT |
| MCIDAS-F | GCTGACTGCTCTTCGCTACT |
| MCIDAS-R | AGAGAAGGGGAAGTCTCCGC |
| MCOLN1-F | CGGATGACACCTTCGCAGC CTAC |
| MCOLN1-R | CGGATGACACCTTCGCAGCCTA C |
| NR4A1-F | AGCATTATGGTGTCCGCACAT |
| NR4A1-R | TTGGCGTTTTTCTGCACTGT |
| RPP25-F | CCTCCTGATACCCTTGAGATGC |
| RPP25-R | TGAGAACACTACATGCCGAGC |
| SLC16A3-F | CCATGCTCTACGGGACAGG |
| SLC16A3-R | GCTTGCTGAAGTAGCGGTT |
| SLC9A3R2-F | GGCCAGTACATCCGCTCTG |
| SLC9A3R2-R | CAGGTCACTTCGGGACGAG |
| TINCR -F | TGTGGCCCAAACTCAGGGATACAT |
| TINCR -R | AGATGACAGTGGCTGGAGTTGTCA |
| TMEM106A-F | AGAGGCTGAAGCCCAAGC |
| TMEM106A-R | GAGGTCACCAGGCAGATGAG |
| TRABD-F | GCAAGAGGGACGTTGTGAAGA |
| TRABD-R | GGACACACGATATTGGCAGAG |
| WNT10A-F | GGCAACCCGTCAGTCTGTCT |
| WNT10A-R | CATTCCCCACCTCCCATCT |
| ZNF385A-F | TGTGCTCTCCCACACTTTTGG |
| ZNF385A-R | TGGCGATTACCTTTGTAGTGC |

| si target | Sense (5’-3’) | Antisense (5’-3’) |
| --- | --- | --- |
| mTOR | CCGCAUUGUCUCUAUCAAGTT | CUUGAUAGAGACAAUGCGGTT |
| RAPTOR | AGGGCCCUGCUACUCGCUUTT | AAGCGAGUAGCAGGGCCCUTT |
| RICTOR | ACUUGUGAAGAAUCGUAUCTT | GAUACGAUUCUUCACAAGUTT |
| 4E-BP1 | GUACCAGGAUCAUCUAUGATT | UCAUAGAUGAUCCUGGUACTT |
| 4E-BP2 | GCAGCUACCUCAUGACUAUTT | AUAGUCAUGAGGUAGCUGCTT |
| DNMT1-1 | GGGACUGUGUCUCUGUUAUTT | AUAACAGAGACACAGUCCCTT |
| DNMT1-2 | GCACCUCAUUUGCCGAAUATT | UAUUCGGCAAAUGAGGUGCTT |
| DNMT1-3 | GAGGCCUAUAAUGCAAAGATT | UCUUUGCAUUAUAGGCCUCTT |
| control | GUUCUCCGAACGUGUCACTT | GUGACACGUUCGGAGAACTT |

**Table S3. List of Antibodies used in Western blot.**

| Target | catalog number | Manufacturers |
| --- | --- | --- |
| DNMT1 | 5032S | Cell Signaling Technology (USA) |
| DNMT3A | 44807S | Cell Signaling Technology (USA) |
| DNMT3B | 72335S | Cell Signaling Technology (USA) |
| GAPDH | 5174 | Cell Signaling Technology (USA) |
| P-S6K | 398S | Cell Signaling Technology (USA) |
| S6K | 9202S | Cell Signaling Technology (USA) |
| 4E-BP1 | 9644S | Cell Signaling Technology (USA) |
| 4E-BP2 | 2845S | Cell Signaling Technology (USA) |
| P-4E-BP1 | 2855S | Cell Signaling Technology (USA) |
| P-4E-BP1 | 9451S | Cell Signaling Technology (USA) |
| P-4E-BP1 | 9455S | Cell Signaling Technology (USA) |
| P-AKT | 13038S | Cell Signaling Technology (USA) |
| AKT | 4691S | Cell Signaling Technology (USA) |
| P-mTOR | 5536S | Cell Signaling Technology (USA) |
| mTOR | 2983S | Cell Signaling Technology (USA) |
| Raptor | 2280S | Cell Signaling Technology (USA) |
| Rictor | 2114S | Cell Signaling Technology (USA) |

Methods of special fragment deletion of DNMT1-5’UTR

DNMT1 5’UTR sequence

>hg19_knownGene_uc010xlc.2 range=chr19:10305576-10305755 5'pad=0 3'pad=0 strand=- repeatMasking=none

GGCTCCGTTCCATCCTTCTGCACAGGGTATCGCCTCTCTCCGTTTGGTACATCCCCTCCTCCCCCACGCCCGGACTGGGGTGGTAGACGCCGCCTCCGCTCATCGCCCCTCCCCATCGGTTTCCGCGCGAAAAGCCGGGGCGCCTGCGCTGCCGCCGCCGCGTCTGCTGAAGCCTCCGAG

Fragment 1：GGCTCCGTTCCATCCTTCTGCACAGGGTATCGCCTCTCTCCGTTT

Fragment 2：GGTACATCCCCTCCTCCCCCACGCCCGGACTGGGGTGGTAGACGC

Fragment 3：CGCCTCCGCTCATCGCCCCTCCCCATCGGTTTCCGCGCGAAAAGC

Fragment 4：CGGGGCGCCTGCGCTGCCGCCGCCGCGTCTGCTGAAGCCTCCGAG

PGL3-control vector

Primer

| F | CTTTTGCAAAAAGCTTGGCTCCGTTCCATCCTTCTGC |
| --- | --- |
| R | TTGGCGTCTTCCATGGCTCGGAGGCTTCAGCAGACG |
| F1 | CTTTTGCAAAAAGCTTGGTACATCCCCTCCTCCCCCA |
| R1 | TTGGCGTCTTCCATGGGCTTTTCGCGCGGAAACCGAT |
| F2 | ACAGGGTATCGCCTCTCTCCGTTTCGCCTCCGCTCATCGCCCCT |
| R2 | AAACGGAGAGAGGCGATACCCTGT |
| F3 | CGGACTGGGGTGGTAGACGCCGGGGCGCCTGCGCTGCCGC |
| R3 | GCGTCTACCACCCCAGTCCG |

Primer pairs:

| del1 | F1+R |
| --- | --- |
| del2 | F2+R2 |
| del3 | F3+R3 |
| del4 | F+R1 |
